# Polymeric Nanoparticles: Exploring the Current Drug Development and Therapeutic Insight of Breast Cancer Treatment and Recommendations

**DOI:** 10.3390/polym13244400

**Published:** 2021-12-15

**Authors:** Ali Sartaj, Zufika Qamar, Farheen Fatima Qizilbash, Annu, Shadab Md, Nabil A. Alhakamy, Sanjula Baboota, Javed Ali

**Affiliations:** 1 Department of Pharmaceutics, School of Pharmaceutical Education and Research, Jamia Hamdard, New Delhi 110062, India; sartaz005@yahoo.com (A.S.); zufikaqhamdard@gmail.com (Z.Q.); farheen.fqz@gmail.com (F.F.Q.); atriannu407@gmail.com (A.); sbaboota@rediffmail.com (S.B.); 2 Department of Pharmaceutics, Faculty of Pharmacy, King Abdulaziz University, Jeddah 21589, Saudi Arabia; shaque@kau.edu.sa (S.M.); nalhakamy@kau.edu.sa (N.A.A.); 3 Center of Excellence for Drug Research & Pharmaceutical Industries, King Abdulaziz University, Jeddah 21589, Saudi Arabia

**Keywords:** breast cancer, chemotherapy, phytoconstituents, polymeric nanoparticles, multi-drug resistance, side effects

## Abstract

This manuscript aims to provide the latest update on polymeric nanoparticle drug delivery system for breast cancer treatment after 2015 and how research-oriented it is based on the available research data. Therefore, the authors have chosen breast cancer which is the most frequent and common reason for mortality in women worldwide. The first-line treatment for breast cancer treatment is chemotherapy, apart from surgery, radiation and hormonal therapy. Chemotherapy is associated with lesser therapeutics and undesirable side effects and hence. In addition, drug resistance affects the therapeutic dose to the target site. Although various nano-based formulations have been developed for effective treatment, the polymeric nanoparticles effectively avoid the lacunae of conventional chemotherapy. There has been an effort made to understand the chemotherapy drugs and their conventional formulation-related problems for better targeting and effective drug delivery for breast cancer treatment. Thus, the polymeric nanoparticles as a strategy overcome the associated problems with resulting dose reduction, enhanced bioavailability, reduced side effects, etc. This present review has compiled the research reports published from 2015 to 2021 from different databases, such as PubMed, Google Scholar, ScienceDirect, which are related to breast cancer treatment in which the drug delivery of numerous chemotherapeutic agents alone or in combination, including phytoconstituents formulated into various polymer-based nanoparticles.

## 1. Introduction

Nowadays, the disease state of cancer is still a considerable problem worldwide. Over 1.8 million new cases and 0.6 million cancer deaths were estimated in the United States in 2020 [1]. Among all cancers, breast cancer is the most frequently found in females and is a common reason for mortality in women worldwide. The first-line treatment, i.e., chemotherapy, is still the first choice for breast cancer treatment in combination with other chemotherapy or phytoconstituents. However, chemotherapy has lots of problems such as enhanced systemic side effects, low ability to penetrate the tumor and hence low pharmacokinetic values. In addition, some of the chemotherapy showed a multi-drug resistance and seemed to be a critical reason for the drug failure treatment [2]. Similarly, most of the phytoconstituents have solubility and stability issues which need to be a high dose to achieve the therapeutic values [3]. To overcome these hurdles of chemotherapy and phytoconstituents, the application of nanotechnology for breast cancer treatment which makes them more efficient and successful treatment with reduced side effects has been widely researched [4]. Nanomaterials are commonly used now for cancer therapy. The application makes them excellent drug carriers for photothermal therapy, photodynamic, immunotherapy, as well as chemotherapy [5].

Currently, several nanoparticulate systems have been investigated for targeted drug delivery in breast cancer, like lipid-based, inorganic-based and polymer-based drug encapsulated or conjugated systems [6]. Over the last couple of years, the development of polymeric nanoparticle systems arose drastically for specific and targeted drug delivery in breast cancer treatment approaches. The characterization of polymeric is very critical as a slight change in any parameters can have a detrimental effect or, worse, such as an increase in toxicity. Therefore, small changes could have a devastating effect. Hence, rigorous critical characterization, such as physiochemical, biological, and morphological, will be a necessity for the successful clinical application of polymer-based nanoparticles for breast cancer treatment [7]. The physiochemical properties of polymer-like polymer size, surface charge and hydrophobicity of polymer may have an influence on blood circulation time, biological compatibility and solubility of the polymer during the formulation of nanoparticles, respectively. The sensitivity of polymers also plays a critical role in better therapeutic effects for targeted drug delivery like thermal sensitive, pH-sensitive, mechanical or photosensitive polymers, which release the drug from the polymer when respective stimuli trigger at the targeted site of action. Other properties like biological aspects of polymer and how it affects its biodistribution, clearance and immunogenicity of polymer towards the body system should also be characterized. Indirectly, this biological characterization leads to the biocompatibility concern, which should be characterized before the application of polymers into nanoparticles formulation. The third characterization is the morphological characterization of polymerlike whether the polymer is branched or linear chain affects its solubility of polymers which determine the drug entrapment and hence better therapeutic efficacy. Therefore, the above characterization parameters are related to the selection of polymers which can be used for better and effective delivery of chemotherapy to the target sites for the breast cancer treatment” [8,9]. The delivery of multiple agents through polymeric nanoparticles gives synergistic or additive therapeutic effects and also overcomes the drug resistance of single agents (chemotherapy) through down-regulation of the uptake mechanism. Therefore, these polymeric nanoparticle systems give an alternative pathway for better drug targeting [10]. The polymeric nanoparticle systems have been reviewed in this article. The data compilation has been done for recent research on breast cancer from the last 5 years. The details of single or combination of chemotherapy drugs or combination with phytoconstituents in polymeric systems have been elaborated in this article.

## 2. Methodology

### 2.1. Literature Data Search

The literature review has been retrieved from different databases such as PubMed, Google Scholar, and ScienceDirect from 2015 to 2021 for the compilation of data related to breast cancer treatment using keywords “Breast cancer” and “Chemotherapy” and “Polymeric nanoparticles”.

### 2.2. Study Design Protocol

The inclusion criteria and exclusion criteria have been added to filter the data related to the topic selected. Inclusion criteria selected are English language, Breast cancer treatment, Polymeric nanoparticles, Chemotherapeutics and Phytoconstituents, Combination approaches and pharmacokinetic data or in vitro cell line study. The exclusion criteria selected are pharmacodynamic data, poster presentation, clinical data, review article data and data availability before 2015.

### 2.3. Data Extracted

The data were extracted from selected articles of relevant study and was mentioned chemotherapeutics treatment, their limitation, polymer used, method of preparation, in vivo or cell lines models and result outcomes. The same criteria were also applied to the chemotherapeutics with phytoconstituents and individual phytoconstituents breast cancer treatment.

## 3. Results

**Figure 1:**
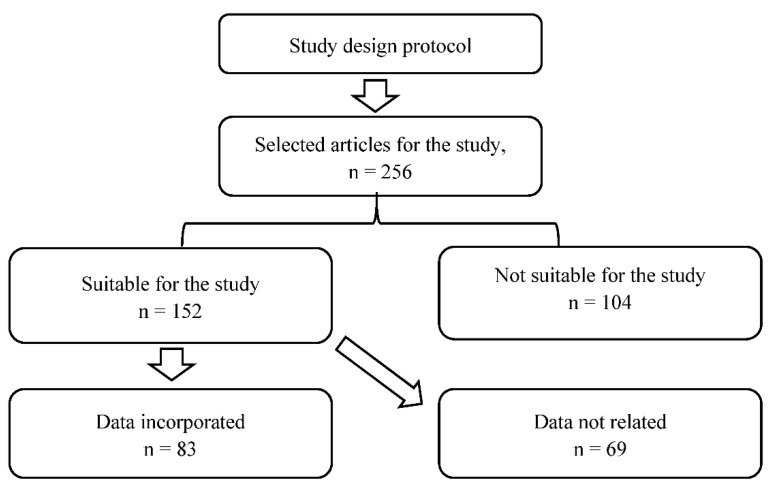
PRISMA protocol used for current study selection criteria.

As per the data suitability of the current manuscript, a total of 256 articles were selected, which were found in electronic media using the selected keyword as mentioned in the literature data search. Among 256 articles, 152 articles were selected which are related to the currently selected topic. Among 152, only 83 articles were selected and investigated as per the above-mentioned inclusion and exclusion criteria mentioned in Section 2.2, which are suitable for the current study, as shown in Figure 1.

## 4. Discussion

The discussion part includes the different research on polymeric nanoparticles of chemo-therapeutic drugs or phytoconstituents or their combination for breast cancer treatment based on the data available after 2015. The discussion part also briefly discusses the drawback of conventional therapy and how they overcome the therapeutic lacuna for better outcomes by using the approach of polymeric nanoparticles, which are summarized in tabular form at the end of each section of the discussion, respectively.

### 4.1. Paclitaxel and Their Combinational Approaches for Breast Cancer Treatment through Polymeric Nanoparticles Drug Delivery System

#### 4.1.1. Paclitaxel (TPGS and PCL Polymers)

This study was carried out by Bernabeu and associates to develop a delivery system by using the ‘Pegylation strategy’ to enhance the biodistribution of the carrier system. There was the development of two block copolymers with poly (caprolactone) loaded with Paclitaxel (PTX). The first NP formulation was composed of alpha-tocopheryl polyethylene glycol 1000 succinate-block-poly (caprolactone) (TPGS-b-PCL-PTX) and another contained methoxy-PEG block-poly (caprolactone) (mPEG-b-PCL-PTX). The in vitro study was carried out among the prepared formulations and the marketed preparation of PTX, i.e., Abraxane^®^, to evaluate the anti-tumor effect on cancerous cell lines (MCF-7 and MDA-MB-231) and it was discovered that TPGS-b-PCL-PTX showed efficient anti-tumor effect of PTX on both the cancer cell lines as compared to other formulations. Moreover, the cellular uptake of PTX was found to be higher at 1.42, 1.85, and 1.6 folds (MCF-7) and 1.3, 2.18, and 2.32 folds (MDA-MB-231) from TPGS-b-PCL-PTX than other formulations. Lastly, the study was concluded by depicting the competent effect of TPGS over PEG and the approach of developing NPs formulation signified the enhanced uptake of PTX by breast cancer cells [11].

#### 4.1.2. Paclitaxel (Chitosan Polymer)

This study was carried out to incapacitate the limitations associated with PTX, such as hydrophobicity and high hemolytic toxicity. Therefore, Gupta and co-workers loaded this anti-cancerous drug into the bio-degradable material, i.e., chitosan (CS), via water in the oil nanoemulsion method. The developed nanoformulation (PTX-CS-NP-10, which is a sample code) was subjected to an in-vitro release study to evaluate the amount of PTX released from the NPs and it was observed that almost 60% of PTX was released in 24h. MTT assay was utilized to evaluate the anti-cancerous effect against MDA-MB-231 mammary cancer cell lines; it was discovered IC50 value of PTX-CS-NP-10 was 1.6 folds lower than the free PTX (pure form). The extent and mode of apoptosis by formulations (PTX, blank CS-NP-02, PTX-CS- NP-10) was evaluated using Annexin V and propidium iodide (PI) kits; the results so obtained showed that the % of apoptotic cells treated with PTX-CS-NP-10 were higher than others. Moreover, the late apoptosis examined in the case of PTX-CS-NP-10 was around 2-folds higher than the free PTX, suggesting that the chitosan-based loaded PTX nanoformulation could aid in targeted delivery of the drug and show efficient anti-cancerous action [12].

#### 4.1.3. Paclitaxel and Hyaluronic Acid (Chitosan and PDEGMA Polymers)

Zhang and associates developed a dual responsive novel formulation by combining the pH and temperature sensitive chitosan and poly (di (ethylene glycol) methyl ether meth-acrylate) (PDEGMA), respectively, followed by grafting it with HA to produce HA-CS-g-PDEGMA. Anti-cancerous drug PTX was then loaded into the developed nanoparticle (HA-CS-g-PDEGMA) represented as HA-CS-g- PDEGMA-PTX NPs. The anticancer efficiency of the developed formulation was evaluated by performing in vitro and in vivo studies. MTT assay was used to perform a comparative in vitro cytotoxicity study among free PTX, HA-CS-g-PDEGMA NPs and HA-CS-g-PDEGMA-PTX NPs on MDA-MB-231 tumor cell line while taking HUVEC cells as control. It was observed that HA-CS-g- PDEGMA-PTX NPs as compared to the other two formulations, showed insignificant cytotoxicity in HUVEC cells and higher toxicity in cancerous MDA-MB-231 cells line suggesting the active targeting of HA receptors confined on the surface of the cancerous cell line. The apoptosis of cells in tumor tissue was carried out using a terminal deoxynucleotidyl transferase dUTP nick end labelling assayed by TUNEL kit, and it was discovered that HA-CS-g-PDEGMA-PTX NPs were able to produce an extensive range of apoptosis of tumor cells compared to free PTX. Considering all the results, it was concluded that the developed nano-particle embraced a favorable treatment of mammary tumors [13].

#### 4.1.4. Paclitaxel with Transferrin (Tf)

Transferrin (Tf), popularly known as the iron-requisite plasma glycoprotein, is meant for the transportation of iron through cell surface Tf receptors. These receptors are mainly confined in the flourishing cancerous cells rather than normal tissues. Therefore, the incorporation of Tf in the NPs can result in the enhancement of the cellular uptake of the drug due to the receptor-mediated endocytosis. Moreover, the attachment of magnetic nanoparticles (MNP) to polymeric NPs could upgrade the delivery of the drug at the targeted site with reduced toxicity and enhanced drug effectiveness. Therefore, while utilizing these proper-ties of Tf and MNP, Cu Y and co-worker developed a PLGA nanoformulation loaded with PTX modified with MNP via solid-in-o/w evaporation method, trailed by Tf adsorption on the surface. MCF-7 and U-87 cancerous cell lines were used to discover the cytotoxicity and cellular uptake of the PTX from the developed formulation (Tf-PTX-MNP-PLGA-NPs). It was discovered that there was a heightened cytotoxicity effect and cellular efficiency was found to be upsurged under the influence of the Tf receptor due to the endocytosis as compared to free PTX and unaltered NPs. Additionally, on the application of the magnetic field, the cellular uptake of Tf-PTX-MNP-PLGA-NPs was found to be efficient, but no marked difference was observed in comparison to others. The study concluded that the attachment of Tf to the PLGA-NP could be used further for the treatment of the mammary tumor by the patients [14].

#### 4.1.5. Paclitaxel and Keratin

Taxane, known as Taxon (paclitaxel, PTX), is found to be effective in treating various kinds of malignancies of diverse origins, but the foremost target is mammary cancer. To overcome the drawbacks associated with PTX such as low aqueous solubility, hypersensitivity, hypotension and neurotoxic effect. Foglietta and associates developed a novel formulation by loading PTX into keratin nanoparticles (KER-NPs-PTX). MCF-7 and MDA MB 231 cell lines of human breast cancer were used. The activities were evaluated among free PTX, albumin loaded with PTX (HSA-NPs-PTX) and KER- NPs-PTX. There was a percentage delay (31.1 ± 5.4) in apoptotic cells of MCF-7 by PTX from KER-NPs-PTX as compared to the other free PTX (28.6 ± 5.1) and HSA-NPs-PTX (24.5 ± 6.4) whereas, in case of MDA MB 231 cell lines, opposite change was observed at different time intervals suggesting that KER-NPs-PTX might be further effective in inducing early cell death. On application for p3D model for the same study, it was discovered that only KER-NPs-PTX was able to induce a significant (*p* < 0.05) decrease in the % of living cells (74.0 ± 8.9 at 24 h and 69.0 ± 6.5 at 48 h), followed by a significant (*p* < 0.05) increase in early apoptotic cells (19.0 ± 4.7 at 48 h) in MCF-7 cells as compared to HSA-NPs-PTX that failed to show any effect furthermore, a significant decrease in % live MDA MB 231 cells was observed on getting treated with KER-NPs-PTX, while other two formulations showed negligible effect [15].

#### 4.1.6. Paclitaxel and Estrone

Yang and co-worker developed a novel formulation based on the process of endocytosis induced by Estrone (ES) and intracellular pH-dependent drug release. The formulation was formulated by graft copolymerization of glycol chitosan with 2-(diisopropylamine) ethyl methacrylate (GCNP) followed by conjugating ES. After the development of GCNP-ES, PTX was loaded in the nanoformulation and was labelled as PTX-GCNP-ES. The internalization of the prepared formulation was carried out using MCF-7 and MCF-10A cell lines which showed that the accumulation of GCNP-ES was 3-folds higher in MCF-7 cells endosomes than MCF-10A cells; furthermore, it was discovered that the incorporation of GCNP-ES was 5-folds higher than GCNP in the cells. On performing cytotoxicity and apoptotic study among free PTX, PTX-GCNP, PTX-GCNP-ES, it was revealed that PTX-GCNP-ES possessed higher cytotoxicity and cell apoptosis than GCNP, suggesting that the conjugation of ES enhanced the cellular uptake of NPs, thereby advancing the in vitro anti-tumor effectiveness. Moreover, tumor growth response to prepared NPs was further evaluated, it was observed that the % tumor inhibition rate was higher in the case of PTX-GCNP-ES approx. 81.4%, whereas PTX solution, PTX-GCNP attained 48.4% and 69.4%, respectively. Furthermore, the in vivo study revealed no hematological and histological toxicity by PTX-GCNP-ES. The study was concluded by suggesting that GCNP-ES was able to deliver PTX in the desired concentration for the treatment of mammary tumors [16].

#### 4.1.7. Paclitaxel and Hyaluronic acid (PLGA Polymer)

Cerqueira and associates prepared a novel formulation via modified oil-in-water emulsion method by loading PTX in PLGA (poly (lactic-co-glycolic acid) later coated with HA (HA-PTX-PLGA) to dynamically target triple-negative mammary cancer. The in vitro release study was carried out using MDA-MB-231 cells to demonstrate the release of PTX from HA-PTX-PLGA and HA-uncoated NPs (PTX-PLGA). It was observed that HA-PTX-PLGA NPs improved the cytotoxicity of PTX in the cancerous cell as compared to PTX-PLGA. Furthermore, there was an enhancement in the cellular uptake of PTX from HA-coated NPs, suggesting that the interface between HA and CD44 resulted in the receptor facilitated endocytosis. Moreover, the hemolytic property was assessed to ensure that no complications would arise during intravenous (i.v.) administration. It was observed that HA-PTX-PLGA NPs did not show any hemolysis suggesting that the prepared formulation was appropriate for i.v. administration. The study was then concluded by signifying that the prepared NPs were effective in showing the anti-cancerous effect towards triple-negative mammary cancer [17].

#### 4.1.8. Paclitaxel and Salinomycin

Combinational drug therapy is supposed to provide the synergistic effect of drugs and targeted delivery with the enhanced therapeutic effect of dual drugs [18]. Therefore, to evaluate the synergistic effect of Salinomycin (SLM) and PTX against the mammary tumor, Muntimadugu et al. developed a nanoformulation by loading SLM and PTX in poly (lactic-co-glycolic acid) nanoparticle employing emulsion solvent diffusion method via cationic stabilizers. SLM was incorporated for targeting cancer stem cells (CSCs) whereas, PTX was utilized for killing cancer cells. An in vitro cytotoxicity study was carried out on MCF-7 cell line amongst combinations formulations, i.e., SLM-HA-NPs + PTX-NPs showed 88.91 ± 2.01% cytotoxicity and it was discovered that the prepared NPs were more efficient than the SLM + PTX solutions which showed only 34% cytotoxicity. Cellular uptake efficacy was evaluated qualitatively and quantitively by loading NPs into fluorescein isothiocyanate isomer (FITC) (FITC-NP, HA-FITC-NP) in which it was detected that there was a 1.5-folds enhanced increase in HA-FITC-NPs than FITC-NP. The binding affinity of NPs to CD44 (overexpressed on CSCs) was performed using MDA-MB-231 cells by means of CD44 human antibody and CD44+ that were tailored using flow cytometry. The attachment of HA to SLM-NPs further enhanced the action of SLM-NPs in inhibiting the growth of CD44 and exhibited enhanced cytotoxicity with low IC50 on cancerous mammary cells. In the case of a combination of nanoparticles, the synergism was observed in the cytotoxicity activity to the growth % of CD44+ cells. Upon performing the pharmacokinetic study, it was observed that there was upgraded bioavailability of the dual drugs from NPs, signifying an extended period of circulation. The study was then concluded by emphasizing the significant effect of combination drug therapy using conventional chemotherapeutic drug candidates in the treatment of mammary cancer and their synergistic effect against the CSCs [19].

#### 4.1.9. Paclitaxel and Gemcitabine

This study was carried out to evaluate the synergistic effect of PTX and Gemcitabine (GEM) for the treatment of breast cancer. To discover this, Dong and co-workers developed PTX and GEM polymeric NP, i.e., MPEG-PLA (methoxy poly (ethylene glycol)-poly (lactide-coglycolide)). An MTT assay was used to assess the in vitro cytotoxicity study among different formulations such as PTX, GEM and their combination (PTX + GEM), using different mammary cancer cell lines (4T1, MCF-7, and MDA-MB-231). It was discovered that the combination could proficiently inverse the resistance of drugs in mammary breast cancer cell lines suggesting that the combining strategy of these two drugs would result in treating breast cancer efficiently and lowering the drug resistance. The in vivo anti-cancer effect, as well as systemic toxicity, was performed on 4T1 tumor-bearing mice amongst phosphate buffer solution (PBS), free PTX, free GEM and PTX–GEM/MPEG– PLA (PG/PP) NPs and it was observed that prepared NPs executed improved anti-cancerous effect and possessed low systemic toxicity. This study was then concluded by suggesting that the combination has a better capability for treating breast cancer than monotherapy [20].

#### 4.1.10. Paclitaxel and Folic Acid

This study was carried out with the motive of targeted delivery of PTX by the development of the lipid-based polymer hybrid composed of FA followed by the further alternation of the lipid shell and polymer core NPs (FLNPs). The complexed NPs were formulated through thin-film hydration and ultrasonic dispersion method. The coating of the single layer of the lipid was analyzed using TEM and dynamic light scattering. EMT6 cell lines were used that highlighted the targeting efficiency of the FA due to the attachment of the folate to the surface of the cancer cell. In-vitro cytotoxicity displayed the cytotoxic effect of the formulation in this order (Taxol^®^ > PTX-FLNPs > PTX-LNPs (without FA). The intertumoral administration of the PTX-FLNPs into the cancerous cells depicted that folate conjugated NPs possessed anti-tumor efficacy similar to the marketed formulation but poor toxicity. However, PTX-FLNPs showed 1.3-folds higher inhibition of the tumor growth than the PTX-LNPs. The study was later concluded by suggesting the advantageous nature of the FA conjugated polymer in treating breast cancer [21]. The docetaxel and their combination polymeric nanoparticle drug delivery approach for breast cancer treatment are summarized in Table 1.

### 4.2. Docetaxel and Their Combinational Approaches for Breast Cancer Treatment through Polymeric Nanoparticles Drug Delivery System

#### 4.2.1. Docetaxel (Magnetic Manganese Oxide)

Multifunctional NPs have a wide range of applications in chemotherapy, image-guided surgery and diagnosis. In regard to the vast application of NPs, a study was conducted in which Abbasi et al. formulated polymeric theragnostic NPs (PTNPs) encapsulating Docetaxel (DTX), a fluorescent dye and magnetic manganese oxide (MnO) for dual chemotherapy and imaging. On performing fluorescence microscopy examination on MDA-MB-231 cell lines, it was observed that there was energy-dependent cellular uptake of DTX followed by prolonged cytoplasmic retention in cancer cell lines. Furthermore, the result obtained from the cytotoxicity study revealed that DTX-loaded PTNPs possessed higher cytotoxicity (almost 80%) than free DTX, with an almost 3–4.4-times decline in the dose of the drug required to achieve cell growth inhibition. Additionally, substantial deposition and extended retention of PTNPs were observed in breast cancer cell lines with the help of fluorescence imaging (FI) and magnetic resonance imaging (MRI). The study was then concluded by suggesting PTNPs could be a promising method to deliver the poorly soluble drug across the breast cancer cells [22].

#### 4.2.2. Docetaxel (PAM-PBLG-b-TPGS Polymers)

Various researchers have revealed that taxane-based chemotherapy has a prodigious ability to treat cancer. To utilize this ability of the taxane group, Wang and associates developed two novel formulations by loading DTX in PAMAM based poly (γ-benzyl-l-glutamate)-bd-α-tocopheryl polyethylene glycol 1000 succinate (PAM-PBLG-b-TPGS) NPs and PAMAM-based poly (γ-benzyl-l-glutamate) (PAM-PBLG) NPs with the nano-precipitation method. On performing a comparative study among all the NPs on cellular uptake efficacy and cell viability using human breast cancer cell line MCF-7 and human cervical cell lines Hela, it was observed that DTX (PAM-PBLG-b-TPGS) NPs displayed stronger cytotoxicity and a 2.5-folds increase in uptake over DTX loaded PAM-PBLG NPs. Moreover, DTX-PAM-PBLG-b-TPGS showed an effective anti-tumor effect and the result found that tumors were of smaller size (final average volume at 200.14 mm^3^) and lighter (final average weight at 75.79 mg). Therefore, the study was concluded by depicting that PAM-PBLG-b-TPGS NPs were very effective for the targeted delivery of chemotherapeutic drugs to treat human breast cancer and cervical cancer [23].

#### 4.2.3. Docetaxel (PLGA-TPGS Polymers)

Multiple drug resistance (MDR) is a major knowledge barrier in achieving success in the treatment of cancer. Thus, to overcome this barrier, an effective nano-based therapy was adopted by Tang et al., who successfully encapsulated DTX in polymeric NPs (PLGA NPs) in addition to an inhibitor of MDR, i.e., Poloxamer 235 followed by conjugation with D-α-tocopheryl PEG 1000 succinate (TPGS) leading to the development of the nanoformulation DTX-PLGA-TPGS/Poloxamer 235 NPs. The capability of the formulation to overcome MDR was assessed in DTX resistant human breast cancer cell lines (MCF-7/TXT). The comparative study among DTX-PLGA- TPGS/Poloxamer 235 and PLGA-TPGS/Poloxamer 235 NPs (PNTPs) showed that there was significant uptake of the DTX loaded NPs than PNTPs in the cancer cell lines. Moreover, marked toxicity was observed in in vitro cell line study with DTX-PLGA-TPGS/Poloxamer 235 NPs (increased by 29.28% (*p* < 0.05) in MCF-7/TXT cells than the marketed form of DTX, suggesting that the developed formulation loaded with DTX and Poloxamer 235 could treat breast cancer significantly [24].

#### 4.2.4. Docetaxel (PLGA)

Triple-negative breast cancer is considered to be the worst type of cancer due to the consequences that occur post-chemotherapy. However, to avoid taxane-resistant triple-negative breast cancer, Bowerman and associates adopted a novel delivery system to deliver the chemotherapeutics drug, i.e., DTX, at the target site. This system involved the fabrication of the Rod-shaped NPs loaded with DTX using an imprint lithography-based technique denoted as Particle Replication in Nonwetting Templates (PRINT). These rod-shaped PLGA-docetaxel nanoparticles tested in the C3(1)-T-antigen (C3Tag) genetically engineered mouse model (GEMM) of breast cancer were used due to their better representation towards the clinical impact of drug entity. The data obtained from the pharmacokinetic study suggested that the developed NPs enhanced the circulation time of DTX, leading to the desired exposure of the DTX to the tumor when compared with the Taxotere^®^. Thereby, the study was concluded by reflecting the noticeable improvement in the therapeutic efficacy of the chemotherapeutic drug delivered via NPs to treat breast cancer [25].

#### 4.2.5. Docetaxel with Folic Acid

**Figure 2:**
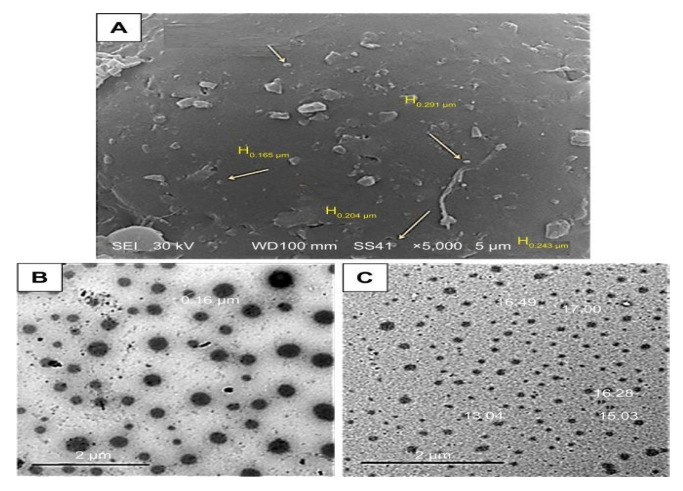
(**A**) SEM photographs of DTX-loaded PLGA NPs design. (**B**,**C**) TEM photographs of PLGA NP design (Taken from open access journal under the terms of creative common attributes) [26].

The complexity of breast cancer is due to the resistance to chemotherapy. To subside this challenge Abou-El-Naga et al. fabricated a polymeric-based nanoformulation (Figure 2) encapsulating DTX using solvent evaporation method followed by conjugation with folic acid (FA). The fabricated formulation DTX-loaded FA/PLGA NPs showed higher cellular uptake efficacy due to the activation of the endocytosis mechanism than DTX-PLGA NPs and free DTX. Additionally, DTX-loaded FA/PLGA NPs restricted the efflux of DTX by declining the expression of ABCG2 by 3.2-folds and MDR1 by 2.86-fold. Moreover, a high apoptotic effect was observed with the formulation conjugated with FA through the activation of TP53 genes, Caspase-9, and Caspase-3, by 1.86-, 2.8- and 1.6- times, respectively [26].

#### 4.2.6. Docetaxel (PHBV Polymer)

Vardhan et al. researched by loading DTX in poly hydroxy butyrate-co-hydroxy valerate (PHBV) NPs via modified emulsification solvent evaporation method. SRB (sulforhodamine B) assay was used to demonstrate the cytotoxicity study between the formulation, free drug and Adriamycin^®^ as a positive control which showed a high percentage of inhibition by NPs than other preparation. The research was then concluded by indicating that NPs could be a better approach for delivering drugs at the target site and to achieve effective extended-release of the drug [27].

#### 4.2.7. Docetaxel (PEG-PLGA Polymers)

Jain et al. encapsulated DTX in PLGA NPs functionalized by estradiol (E2) to achieve targeted delivery of DTX. To support this hypothesis, a cellular uptake study was conducted on ER-positive (MCF-7 cells) and negative (HeLa cells), which showed that the developed formulation (E2-DTX-PEG-PLGA) NPs were taken up in considerable amount in MCF-7 than HeLa cells as compared to the other formulations. Furthermore, MTT assay discovered comparatively advanced cytotoxicity of developed formulation compared to free DTX. In a pharmacokinetic study, it was observed that there was a 9.36- and 4.79- times improvement in the circulation and AUC (0–∞), respectively, of NPs when compared with the marketed formulation. The antitumor efficacy was further demonstrated in a DMBA-induced rat model, which showed that there was a substantial decline in the tumor volume when compared with the simple PLGA-NPs. This research suggested that the functionalization of NPs with E2 could be a better option in treating breast cancer with specific targeting efficacy as compared to the simple PLGA-NPs (without E2) [28].

#### 4.2.8. Docetaxel (Chitosan)

The current study was conducted by Jain and associates involved the development of chitosan nanoparticles ionically cross-linked with DTX using sodium tripolyphosphate (TPP) as a cross-linker. MDA-MB-231 cell lines were used to perform an in vitro cytotoxicity study that exposed the beneficial effects of developed NPs over pure DTX with a nearly 85% reduction in the viability of cells. The study displayed the advantages of ionically cross-linked NPs, further suggesting it as a hopeful drug delivery system for the treatment of breast cancer [28].

#### 4.2.9. Docetaxel (PLG)

Pioneering research was performed in which Yang et al. developed polymer-based hybrid NPs conjugated with dual-ligand for targeted remedy of HER2/neu-overexpressing cells. This innovative nanoformulation (consisted of a poly(lactide-co-glycolide) contained an anti-HER2/neu peptide (AHNP) imitated with an altered HIV-1 TAT (mTAT) in addition to DTX. The data of cellular uptake efficacy displayed that the hybrid NPs transformed the caveolae-mediated endocytosis (CvME) cellular uptake pathway to multiple endocytic pathways leading to the uplifting of NPs incorporation into the cells. Moreover, a high concentration of DTX was reported in SK-BR-3 breast cancer cell line on treatment with DTX-encapsulated dual-ligand hybrid NPs (IC50 value 0.98 ng/mL) than any other preparations due to the overexpression of Her2/neu. The study was concluded by highlighting the significant effect of dual-ligand hybrid NPs against breast cancer in addition to the targeted delivery [29].

#### 4.2.10. Docetaxel and Thymoquinone

Combination chemotherapeutics study by Zafar et al., who optimized lipid nanocapsules grafted with chitosan (CLNCs) for the targeted delivery of DTX and thymoquinone (THQ) against breast cancer. The CS fabricated LNCs enhanced the cellular uptake of the combination therapy, in addition to the endosomal escape effect, leading to substantially advanced cytotoxicity contrary to triple-negative (MDA-MB-231) and MCF-7 cell lines. In addition, a chick embryo chorioallantois membrane (CAM) assay was conducted that presented an improved anti-angiogenic effect with the dual drug-loaded CLNCs (72.2 ± 6.3% at 48 h) than other preparations like DTX and THQ treated groups showed an inhibition of 21.8 ± 6.1% and 22.8 ± 6%, respectively after 24 h of study. The in-hand data suggested developed formulations could be used for targeting breast cancer and inhibiting multiple drug resistivity [30].

#### 4.2.11. Docetaxel and Sulforaphane

A combinatorial study was carried out by Huang and associates against differentiated breast cancer cells (DBCCs) and breast cancer stem cells (BCSCs) by treating them with the DTX and sulforaphane (SFN) combination loaded in PLGA-b-HA NPs. Dual drug-loaded NPs showed a marked effect against both the cell lines in comparison with the free drug. The in vivo study revealed that this combination therapy subdued the self-activating ability of BCSCs. Thus, the study was concluded by highlighting the anti-tumor effect of the combination therapy was found to be more significant (*p* < 0.01) than monotherapy, further signifying the potency of polymeric NPs in targeting DBCCs and BCSCs [31].

#### 4.2.12. Docetaxel and Gemcitabine

Kushwah and associates loaded a combination of DTX and gemcitabine (GEM) in a polymeric-based nano-formulation (DTX-PEG-GEM) to assess their efficacy against breast cancer. The cellular uptake efficacy of the formulation was carried out using coumarin-6 (C-6) dye in MCF-7 and MDA-MB-231 cell lines, which displayed that there was upsurged cellular uptake of C-6 treated NPs via clathrin-facilitated endocytosis. Higher inhibition in the tumor growth was observed in the cells when treated with NPs, in addition to an improved survival rate. Furthermore, less toxicity was observed in the liver and kidney on treatment with NPs than the market formulations of DTX, i.e., Taxotere^®^ and GEM, i.e., Gemzar^®^. The compiled data of the research claim improved therapeutic efficiency and decreased toxicity of the dual drug-loaded polymeric NPs. AUC (0–∞) value of each drug DTX and GEM also increased by 5.38 and 4.82-fold, respectively, in dual-loaded NPs as compared to Taxotere^®^ and Gemzar^®^ [32].

#### 4.2.13. Docetaxel and Salinomycin

**Figure 3:**
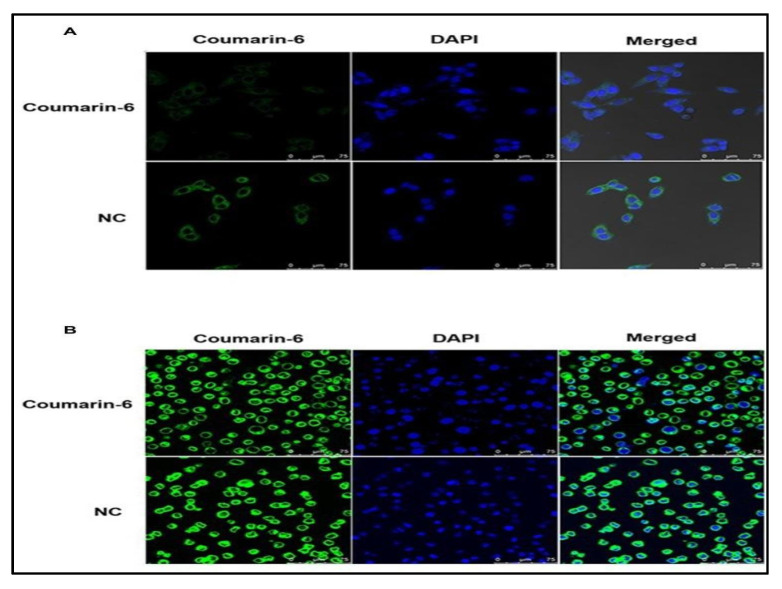
In vitro cellular uptake of nanoparticles. MCF-7 cells (**A**) and MCF-7-MS (**B**) were treated with coumarin-6 and NC after 1 h, followed by staining with DAPI for nuclei. The green fluorescence of coumarin-6 and blue fluorescence of DAPI were analyzed by a confocal laser scanning microscopy (Taken from open access journal under the terms of creative common attributes) [33].

Gao and co-workers developed a nanoformulation by loading paclitaxel (PTX) and salinomycin (SLM) in the PLGA/TPGS (Tocopheryl polyethylene glycol 1000 succinate) nanoparticle via nanoprecipitation method. Different ratios and the concentration of SLM and DTX were optimized to obtain synergistic effect by performing in-vitro cytotoxicity in MCF-7 cells and MCF-7-MS. It was observed that SLM was more prone to show cytotoxicity in MCF-7-MS than MCF-7 cells whereas, DTX showed the opposite effect. However, in combination, the effect was found to be higher in both the cell lines, as shown in Figure 3. On performing in vivo studies, it was observed that the SLM + DTX NP was able to extend the circulation time (2.2 folds increased plasma retention compared to DTX+SLM) thereby, displaying higher targeting efficiency and anti-tumor effect in contrast to other treatments. Later, the study was concluded by depicting that the prepared nanoformulation (SLM +DTX PLGA/TPGS) was able to show a synergistic effect in in vitro and in vivo studies, consequently providing a superior strategy for the reduction of breast cancer in combination [33].

#### 4.2.14. Docetaxel and Hyaluronic Acid

DTX shows its mechanism of action by inhibiting tubulin polymerization and decreasing the expression of BCL-2 genes. So, Mirzaie et al. developed a formulation by conjugating DTX with chitosan (CS) loaded in NPs in the presence of hyaluronic acid using the spontaneous ionotropic gelation method. The effects of DTX-Cs-HA NPs were evaluated on the BAX and BCL-2 genes expression on MCF-7 cell lines using real-time PCR, which showed that gene expressions significantly decreased in the cells being treated with NPs rather than free DTX. In vitro cytotoxicity study revealed that a longer duration of the treatment by NPs effectively increased the cell viability in human fibroblast cell lines and IC50 values of free DTX and CS -DTX NPs using human fibroblast cell after 72 h was found 0.368 ± 0.21 μg/mL, and 23.139 ± 0.35 μg/mL. These efficacious results of the NPs suggested that conjugation of DTX with NPs can be a better approach for the treatment of breast cancer [34]. The docetaxel and their combination polymeric nanoparticle drug delivery approach for breast cancer treatment are summarized in Table 2.

### 4.3. Polymeric Nanoparticles of Doxorubicin and Their Combinational Drug Delivery System for Breast Cancer Treatment

#### 4.3.1. Doxorubicin (PLGA-PLL and PLA-PEG Polymers)

In this study, the Doxorubicin (DOX) was loaded into polymeric nanoparticles, which showed in vitro drug release for 30 days. The cytocompatibility of drug-free polymer, i.e., PLGA-b-PLL and PLA-b-PEG and growth inhibition of drug-loaded polymeric nanoparticles were investigated on MDA-MB-231 breast cancer cells. The luminescence cell viability assay showed that minimal toxicity even at 0.2–2000 µg/mL concentration of drug-free nanoparticles, while it showed reduced cell viability when DOX-loaded nanoparticles of >10 µM concentration were used [35].

#### 4.3.2. Doxorubicin (mPEG-PLGA Polymers)

In this study, DOX is used for brain metastatic breast cancer, which is most challenging to treat because of the blood-brain barrier (BBB) and another hurdle is the lack of targeted drug delivery. A study of polymeric nanoparticles was used in brain metastatic breast cancer cells (MDA-MB-831); the small size and anion surface of polymeric particles make its high penetration and retention in the brain compared to the PEGylated nanoparticle alone. In vitro study showed high preferential cytotoxicity when DOX polymeric particles were used. The extended systemic circulation and retention of polymeric nanoparticles compared to uncoated nanoparticles were also confirmed by ex vivo near-infrared imaging on nude mice [36].

#### 4.3.3. Doxorubicin (PEG Polymer)

To the targeted drug delivery in folate receptors overexpressing breast cancer, DOX loaded into PEG-modified gold nanoparticles investigated in this research which was augmented by laser photothermal therapy. MDA-MB-231 breast cancer cells used for in vitro studies showed the better therapeutic efficacy of combinational therapy compared to the free DOX and also it showed effective results compared to MCF-7 cells in which the expression of a low level of folate receptors was found. Hence this system of folate binds with doxorubicin-loaded into gold nanoparticles are a captivating platform for targeted drug delivery which is suitable for photothermal cancer therapy [37].

#### 4.3.4. Doxorubicin (PLGA and DSPE-PEG 2000 Polymers)

**Figure 4:**
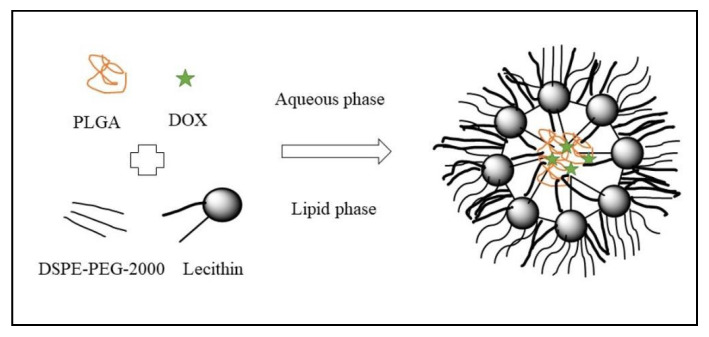
Schematic diagram of the DOX NPs (Reproduced from open access journal under the terms of creative commons attributes) [38].

The nanoprecipitation method was used for the preparation of hybrid nanoparticles made of lipid and polymer for controlled delivery of DOX. The structural components of hybrid nanoparticles were made of PLGA, lecithin, and DSPE-PEG 2000 as shown in Figure 4. The safety, biocompatibility and particle permeation were supported by in vitro cytotoxicity and confocal imaging. The hybrid nanoparticles showed higher anti-proliferation in MDA-MB231 and PC3 cell lines. Therefore, this strategy provides safe and controlled drug delivery [38].

#### 4.3.5. Doxorubicin (Chitosan Polymer)

In this study, DOX was loaded into thermo-sensitive PNVCL-chitosan nanoparticles. This polymer was further added with a cell-penetrating peptide (CPP) which modified the DOX release. The pH and temperature change in the tumor environment triggers the release of the drug into the tumor cells and the inclusion of CPP which modified the DOX release at the targeted site. Another study in vitro and in vivo showed reduced cytotoxicity in normal cells as compared to DOX alone. In vivo study in TNB xenograft mouse and their result indicated a decrease in tumor volume without any systemic toxicity and hence extended life span [39].

#### 4.3.6. Doxorubicin (O-Succinyl Chitosan and Pluronic^®^ Polymers)

The overexpression of HER2- in breast cancer can be overcome by developing DOX encapsulated into anti-HER2- monoclonal antibodies decorated O-succinyl chitosan graft Pluronic^®^ F127 (OCP) copolymer nanoparticles. The in vitro cytotoxicity of the same showed the lowest IC50 value, which suggests the increase in therapeutic efficacy in HER2 overexpressed human breast cancer and hence developed a promising strategy for HER2- overexpressed breast cancer [40].

#### 4.3.7. Doxorubicin and Indocyanine Green (ICG)

DOX and hyperthermia agent indocyanine green (ICG) combined therapy was developed for breast cancer using folic acid conjugated PCL-PEG-PCL nanoparticles. The thin-film hydration and ultrasonic dispersion method were used for nanoparticle preparation. The surface decoration is done by folic acid conjugation against the folate receptors overexpressing breast cancer. This conjugation improved the uptake of nanoparticles in EMT-6 breast cancer cell lines. Ex vivo fluorescence imaging also confirmed the accumulation of the drug in breast tumors because folate conjugation is given a targeted drug delivery [41].

#### 4.3.8. Doxorubicin and Celecoxib (CXB)

The multi-drug resistance is the most common cause for reducing the intracellular accumulation of various chemotherapeutic drugs by overexpressing P-glycoprotein (P-gp), which is an ATP-binding cassette efflux transporter. In this study, CXB is a COX-2 inhibitor used to enhance the cytotoxicity of DOX in breast cancer overexpressed by P-gp. The drug-resistant breast cancer cell lines MCF-7/ADR cells showed enhanced cellular uptake of HPPDC nanoparticles by CD44/HA-specific endocytosis. COX-2 inhibitors also help to reduce the P-gp expression and hence increase the cytotoxicity and apoptosis in breast cancer cells related to DOX [42].

#### 4.3.9. Doxorubicin and Curcumin (DOX-CUR-PEG-PLGA-PLGlu Polymers)

In this study, a pH-sensitive nanoparticle was developed for DOX and CUR using monomethoxy PEG- b-PLGA-b-P (L-glutamic acid) polymer. The in vitro drug release profile in pH 7.4, which suggested the faster and slower release of curcumin and DOX, respectively. The in vitro study confirmed the anti- tumor effect of DOX-CUR nanoparticles on cancer stem cells (CSCs)-enriching MCF-7/ADR mammospheres. The result showed a decrease in % of CSCs from 39.9% to 6.82% in the control group to the treatment group, respectively, with polymeric nanoparticles of DOX-CUR, and that it could be a useful therapeutic strategy [43].

#### 4.3.10. Doxorubicin and Curcumin- (PEG Polymer)

Transferrin decorated nanoparticles to deliver DOX and CUR for the treatment of breast cancer were investigated in this research. Both drugs are loaded into pH-sensitive polymers of poly (ethylene glycol) and formed formulation Tf-PEG-CUR/DOX NPs. The in vitro cytotoxicity and in vivo antitumor activity suggested that the combination of two drugs exhibited higher cytotoxicity compared to the Tf-PEG-CUR NPs alone [44].

#### 4.3.11. Doxorubicin and Curcumin-Hydroxyapatite Polymer

DOX and CUR are simply co-loaded into a polymer made of hydroxyapatite (HAp) shell and core of iron oxide, which act as the magnetic core. These polymers are loaded with drugs prepared by the simple diffusion deposition method. The in vitro characterization study suggested that the combination approach could inhibit the growth of MCF-7 breast cancer cell lines and HEpG2 liver cancer cell lines. The combination approach was more potent compared to the free drug in respect of dose and duration of action. Hemolysis study confirmed the approach is non-toxic towards the normal cells [45].

#### 4.3.12. Doxorubicin, 5-Fluorouracil and Cisplatin

Doxorubicin, 5-fluorouracil, and Cisplatin are drugs that have poor water-solubility and stability issues. So, in this study, all three drugs are loaded into polymeric nanoparticles of polycaprolactone- polyethylene glycol (PCL-PEG) nanoparticles by double emulsion method ameliorate the solubility and stability of drugs. The MTT assay confirmed the enriched cytotoxicity and effective drug delivery system of polymeric nanoparticles in T47D and MCF7 breast cancer cells. The IC50 value of DOX was lower compared to the remaining two drugs. However, the IC50 values of the drugs were found lower in T47D compared to the MCF-7 cell lines [46].

#### 4.3.13. Doxorubicin and Noscapine (NOS)

DOX and NOS loaded into polymeric nanoparticles by nanoprecipitation method in which the mPEG, PLGA polymeric nanoparticles precipitated with the drug moieties. The NOS nanoparticles and in combination with DOX-HCl were investigated on 4T1 breast cancer cell lines. The result showed that synergistic effects NOS nanoparticles with DOX-HCl and also inhabited the tumor growth by 68.50% more significantly compared to the DOX-HCl, which showed 32%, and NOS nanoparticles which showed 55.10% alone [47].

#### 4.3.14. Doxorubicin and Ag

**Figure 5:**
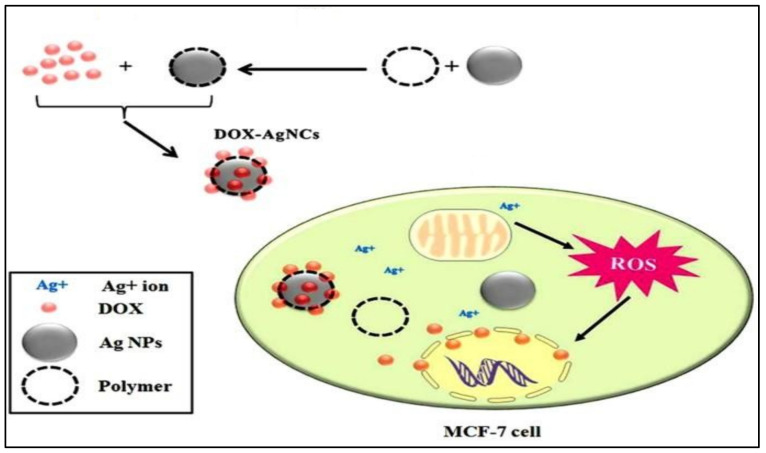
Schematic illustration of the possible mechanism of action behind the resultant synergic cytotoxic effect of DOX-loaded Ag/polymeric NCs on MCF-7 cells (Taken from open access journal under the terms of creative common attributes) [48].

The current study investigated three different polymers, i.e., PVA, PEG, and PVP, with a core of Ag, as represented in Figure 5. The in vitro study was conducted on MCF-7 and human fibroblast (1BR hTERT) cell lines show cytotoxic effects. The Ag and DOX-Ag nanoparticle with different polymers showed more cytotoxicity compared to the normal fibroblasts. The low dose of DOX into the core-shell showed a synergistic effect, with DOX-Ag/PVP being more cytotoxic. Hence, the combinatorial therapy showed enhanced cytotoxicity in breast cancer cells [48].

#### 4.3.15. Doxorubicin and Cisplatin

In this study, the HER2 (Human epidermal growth factor receptor 2) decorated nanoparticle made of aldehyde hyaluronic acid (AHA) and hydroxyethyl chitosan (HECS) to deliver DOX and cisplatin for the synergistic targeted combination was investigated. The in vitro study of both combination drug therapy in MCF-7 breast cancer cell lines showed synergistic cell killing effects and hence novel formulations have promising chemotherapy for breast cancer [49].

#### 4.3.16. Doxorubicin, Chlorin e6 (Ce6), and Colloidal Manganese Dioxide (MnO_2_)

The combination of DOX, Ce6, and MnO_2_ with the polymer of poly (ε-caprolactone-co-lactide)-b-PEG-b-poly(ε-caprolactone-co-lactide) for treatment of breast cancer was investigated. So, these assemble all molecules into a polymer called oxygen-generating theragnostic nanoparticles as MnO_2_ decomposes the excessive H_2_O_2_ in the tumor and acts as tumor hypoxia. Overall, the effect of all with DOX can promote the chemotherapeutic efficacy of polymeric nanoparticles formulation in MCF-7 tumor-bearing mouse model [50].

#### 4.3.17. Doxorubicin and Pyrrolidine Dithiocarbamate (PDTC)

DOX along PDTC poly (ortho ester urethanes) nanoparticles were used in combination to reverse the multi-drug resistance due to P-gp expression by down-regulation and enhanced the drug accumulation in tumor cells. Because of the pH sensitivity of polymers, drugs are released in mild acidic conditions due to ortho easter bond breakage. Monolayer cultured cells (2D) and multicellular spheroids (3D) gave higher cytotoxicity and cell death by DOX in cell lines of MCF-7 and MCF-7/ADR. The in vivo result also suggested enhanced DOX accumulation and higher tumor growth inhibition (82.9%) compared to free drugs [51].

#### 4.3.18. Doxorubicin and Quercetin

Herein the development of biotin-conjugated with PEG-b-PCL nanoparticles encapsulated the DOX and quercetin (chemosensitizer) have been investigated. This approach investigated reverse the multi drug resistance and the study showed the result less P-gp efflux by DOX resistance cells lines (MCF-7/ADR) as compared to the plan drugs or single drug polymeric nanoparticles or without biotin-conjugated polymeric nanoparticles. Overall, this study has a potential role in the effective treatment of breast cancer-resistant drugs [52].

#### 4.3.19. Doxorubicin and Disulfiram (DSF)

Co-loaded delivery of DOX and DSF using amphiphilic polymer PCL-b-PGlu-g-mPEG was investigated. The structure of the amphiphilic polymer is made of a hydrophobic core of PCL and poly (glutamic acid) shell for DSF and DOX, respectively. The polymeric nanoparticle improved the intracellular accumulation as compared with the free drug solution. The synergistic cytotoxic effect was observed in cell lines of MDA-MB-231 and MCF-7. In vivo study also supports the enhanced antitumor activity as compared to the plain drug combination [53].

#### 4.3.20. Doxorubicin and Metformin (MET)

The DOX and MET are loaded into polymeric nanoparticles composed of PLGA and TPGS prepared by the double emulsion method. TPGS inhibits the P-gp efflux and reverses the multi-drug resistance (MDR) of the chemotherapeutics drug. The co-loaded DOX and MET into polymeric nanoparticles showed improved cytotoxicity and cell death in MCF-7/DOX cell lines compared to the plain drugs. The higher cytotoxicity is attributed to the enhanced intracellular drug accumulation in tumor cells. Hence, this strategy could be effectively used to overcome the MDR in breast cancer chemotherapy [54]. The doxorubicin and their combination polymeric nanoparticle drug delivery approach for breast cancer treatment are summarized in Table 3.

### 4.4. Methotrexate and Their Combinational Approach for Breast Cancer Treatment through Polymeric Nanoparticles Drug Delivery System

#### 4.4.1. Methotrexate (Chitosan)

Methotrexate (MTX) is an efficient agent that is used to treat various tumors and autoimmune disorders. The present study was carried out to design a potent delivery nanocarrier of MTX to improve biodistribution and stability, enhance clinical effectiveness, and diminish the adverse effects of the drug. The synthesis of magnetite nanoparticles (Fe_3_O_4_-NPs) was done with the help of Pterocladiella. MTX was encapsulated in CS-Fe3O4-NPs, while 39.8% of the encapsulated drug was first released and then the rest of the amount of the drug was released till 120 h. The IC50 of MTX/CS-Fe_3_O_4_-NPs and MTX was found to be 9.7 and 51.4 μg/mL, respectively, after a period of 72 h. It was seen that the MTX/CS-Fe_3_O_4_-NPs showed destruction of the membrane in the cells of MCF-7 that led to enhancement of the activity of LDH by 5-folds as compared to that of MTX when treated once. On treatment with MTX/CS-Fe_3_O_4_-NPs, it displayed up-regulation of caspase3 and DHFR gene expression. Thus, the MTX encapsulated chitosan-coated Fe_3_O_4_-NPs was seen to display the anticancer activity of MTX as a potent drug for the effective treatment of breast cancer [55].

#### 4.4.2. Methotrexate (PLGA)

Methotrexate (MTX) is an anticancer medicament that is used to prevent the dihydrofolate reductase enzyme that blocks the synthesis of DNA, proteins, and RNA that have poor aqueous solubility. The stability and solubility of medicaments used in delivery systems were improved using MTX encapsulated PLGAbeta-cyclodextrin nanoparticles that were synthesized using a procedure called the double emulsion method. TD7D breast cancer lines were treated with equal amounts of MTX encapsulated PLGAbeta-cyclodextrin nanoparticles and free MTX was treated on T47D breast cancer lines. Whereas the MTT assay verified that the methotrexate-loaded PLGA- beta-cyclodextrin nanoparticles were responsible for the cytotoxicity and proficient drug delivery in the T47D cells of breast cancer. Thus, the results displayed that the loaded drug would prove to be potent in controlled drug delivery for a long period for the effective treatment of breast cancer [56].

#### 4.4.3. Methotrexate and Aceclofenac

The current study was performed to load methotrexate (MTX) and aceclofenac (ACL) in fucose anchored lipid-polymer hybrid nanoparticles (Fu-LPHNPs) that were aimed to attain controlled and targeted delivery for breast cancer treatment. The study aimed to determine the therapeutic effects of the MTX and ACL via LPHNPs in MCF-7 and triple-negative breast cancer cells (MDA-MB-231). The study displayed steady internalization within 3 h of incubation on administration with the coumarin-6 LPHNP formulations with MCF-7 and MDA-MB-231 cells. The DMBA-induced experimental breast cancer mouse model was used for determining the antitumor activity of MTX and ACL-loaded LPHNPs that displayed better results for tumor growth with MTX- and ACL-loaded LPHNPs as compared to the mixture of MTX and ACL or MTX alone. At a higher dose (10 mg/kg) in a DMBA-induced mouse model, the ACL-loaded LPHNPs displayed anticancer and prophylactic properties. Thus, it was concluded that the ACL-LPHNPs confirm the synergistic and anticancer properties in combination with MTX [57].

### 4.5. Platinum Compound and Their Combinational Approach for Breast Cancer Treatment through Polymeric Nanoparticles Drug Delivery System

#### 4.5.1. Carboplatin

The study aimed to synthesize chitosan nanoparticles by the process of the ionic interaction method. The nanocarboplatin was seen to exhibit better compatibility when compared to chitosan nanoparticles. The cytotoxic study of the carboplatin encapsulated chitosan nanoparticles was performed against breast cancer (MCF-7) cell lines that proved that the chitosan nanoparticles could be used as a favorable approach for the effective delivery of breast cancer treatment [58].

#### 4.5.2. Cisplatin (Chitosan)

In one study, it was revealed that the Cisplatin encapsulated iron oxide nanoparticles (IONPs), when reformed with chitosan, could employ cytotoxic effects in human breast cancer cell lines (MDA-MB-231). An extract of eucalyptus leaf was used to synthesize IONPs that was also used as a stabilizing and reducing agent. In this study, the cells of MDA-MB-231 were treated with various ratios of cisplatin, cisplatin-IONPs, and cisplatin-IONPs-chitosan for 24 h. The results showed a higher amount of cytotoxicity as compared to that of free drugs in MDA-MB-231 cells on treatment with cisplatin-IONPs and cisplatin-IONPs-chitosan. While the process of flow cytometry confirmed the apoptosis that exhibited that the amount of apoptosis in the cells that were treated with the dual therapy of cisplatin-IONPs-chitosan were higher with cisplatin alone and cisplatin IONPs [59].

#### 4.5.3. Cisplatin (Dextran)

Another study was performed in the 4T1 orthotropic mammary tumor metastasis model that was treated with cisplatin encapsulated LHRH-modified dextran nanoparticles (Dex-SA-CDDP-LHRH). It was observed that the use of the LHRH ligand was able to maintain the specific targeting efficiency that was bound to the LHRH receptors as they are known to be overexpressed on 4T1 cells of breast cancer. Thus, it was concluded that the Dex-SA-CDDP-LHRH nanoparticles displayed an improved cellular uptake and also led to enhanced cytotoxicity as compared to that of Dex-SA-CDDP nanoparticles (without ligand). However, it was seen that Dex-SA-CDDP-LHRH, as well as Dex-SA-CDDP dis-, played a diminished CDDP’s toxicity and an enhanced CDDP’s tolerance. It was observed that the Dex- SA-CDDP-LHRH led to an increased deposition of CDDP at the organs filled with metastasis and at the primary tumor site, whereas it caused a comprehensive decreased CDDP’s nephrotoxicity. Thus, the study proved that the nanoparticles of Dex-SA-CDDP-LHRH can prove to be a better candidate for the treatment of breast cancer as a targeted chemotherapy approach [60].

### 4.6. 5-FU and Their Combination Approach for Breast Cancer Treatment through Polymeric Nanoparticles Drug Delivery System

#### 4.6.1. 5-Fluorouracil (PLGA)

5-FU displayed various demerits such as non-selective biodistribution, short half-life, and the expansion of the drug resistance criteria via cells of the tumor. The study inspected the usage of folic acid-decorated and PEGylated poly (D, L-lactide-co-glycolide) nanoparticles (FOL-PEG-PLGA NPs) in order to attain specific and targeted delivery of 5-FU for the cancerous breast cancer cells. The nanoparticles of FOL-PEG-PLGA and PEG-PLGA were synthesized using nanoprecipitation that was carried out under optimum conditions and were found to be hemocompatible and thus displayed no cytotoxicity in normal and tumor human breast cancer cell lines viz, CCD-18 and MCF-10A and HT-29 and MCF-7. The in vitro cytotoxicity studies displayed that the cells of breast cancer showed half-maximal inhibitory concentration; that is, the IC50 value for the 5-FU-loaded FOL-PEG-PLGA NPs was approx. 4-fold lesser than the 5-FU-loaded PLGA NPs (*p* < 0.05). Thus, it was concluded that the FOL-PEG-PLGA NPs could prove to be better carriers that can be used as a specific and targeted approach of 5-FU for the treatment of breast and colon cancer [61].

#### 4.6.2. 5-Fluorouracil and Taribavirin

Since the 1990s, the p38MAPK signaling pathway has been explored for the aid of 5-fluorouracil (5-FU) against the treatment of breast cancer. Riavarin, known to be a derivative of nucleotide, is a prodrug of taribavirin (TBV) was lately prepared to have a little concentration in the red blood cells. The 5-FU-linked SLNS and TBV were later synthesized and characterized. The molecular docking studies revealed that the TB and 5-FU-linked SLNs were able to dock the p38MAPK protein successfully. The results showed that the nanoformulation of TBV displayed the highest cytotoxic effects with an IC50 value of about 0.690 μM after 24 h, whereas the free TBV formulation was seen to display an IC50 value of about 0.756 μM [62].

### 4.7. Gemcitabine and Their Combination Approach for Breast Cancer Treatment through Polymeric Nanoparticles Drug Delivery System

#### 4.7.1. Gemcitabine (Fucoidan, and Chitosan)

The NPs were synthesized by polyelectrolyte complexation using two marine-origin polymers viz, fucoidan, and chitosan for the delivery of Gemcitabine (Gem) that is an antitumor medicament. The cytotoxicity studies were performed on MDA-MB-231, EA.hy926 cell line revealed that the Gem encapsulated NPs displayed enhanced toxicity of about 25% as compared to that with the free Gem. The medicament-loaded NPs thus displayed enhanced toxicity without causing any adverse effects against the human cancer cells [63].

#### 4.7.2. Gemcitabine (Chitosan)

The wide therapeutic effectiveness of this medicament is yet to be discovered. It is known to have repeated dosing necessities and low oral bioavailability. In this study, estimation of trimethyl chitosan (TMC)-CSKSSDYQC (CSK) peptide conjugates were analyzed for the enhancement of the oral bioavailability of gemcitabine to target the goblet cells of the intestine and also to increase the cellular uptake. The drug that is, gemcitabine was loaded into CSK-TMC liked conjugates that displayed an enhanced amount of drug permeation via the intestinal epithelial membranes of porcine when compared to that of unconjugated nanoparticles of TMC, whereas the result of the cellular uptake of the conjugates of the drug on HT29-MTX-E12 goblet cells of intestine was found to be both concentration and time-dependent. It was seen that the effluxes caused by multiple resistance protein-2 (MRP2) and P-glycoprotein (P-gp) were able to affect cellular transport. While the pharmacokinetic studies showed that oral bioavailability of about 54% was seen when the TMC loaded NPs were administered as compared to that with a free solution of gemcitabine (9.9%). Moreover, the CSK-TMC conjugated was seen to enhance the oral bioavailability of 60.1% with approx. 5.1-fold increase, thereby diminishing the proliferation of tumor by 3.3-folds in BALB/c nude mouse model as related to that of control group and free gemcitabine solution group [64].

### 4.8. CDK4/6 Targeted Based Drug for Breast Cancer Treatment through Polymeric Nanoparticles Drug Delivery System

#### 4.8.1. Dasatinib

Dasatinib (DAS) is assessed by its low and high pH solubility that makes it troublesome in proving its efficacy in the tumor. In this study, a biocompatible and biodegradable polyester was analyzed for the synthesis of polymeric nanoparticles (NPs). DAS was assessed for its blood compatibility and cytotoxic studies. The biocompatible and biodegradable polyester was aided for the preparation and loading of DAS nanoparticles and the in vitro data that displayed rapid release of DAS as compared to that with Polylactide (the FDA-approved biopolymer). The results showed that the DAS encapsulated polymeric nanocarrier was seen to exhibit superior effectiveness as compared to that of free DAS [65].

#### 4.8.2. Lapatinib

The particular study was carried out to estimate the therapeutic potential of a polymer-lipid hybrid nanoparticle for the effective delivery of lapatinib (LPT) in the treatment of breast cancer. A polymeric core (poly[lactide-co-glycolide]-D-a-tocopheryl polyethylene glycol 1000 succinate [PLGA-TPGS]) was used for the synthesis of the nanoparticles that were later covered using a PEGylated lipid layer (DSPE-PEG) (PLPT) to retain the structural integrity of the formulation. The results revealed that the PLPT was able to destroy the MCF-7 cancerous cells in a concentration and time-dependent way with a proficient cellular internalization. Furthermore, LPT encapsulated nanoparticles were able to stimulate apoptosis in the cancerous cells as compared to that of free LPT. At the same time, the pharmacokinetic study indicated that the nanoparticles led to a comprehensive decline in the reticuloendothelial system (RES) uptake as well as an enhancement in the blood circulation time of LPT. The enhanced circulation period led to the deposition of the drug at the desired site (tumor site). Finally, it was concluded that the PLPT was able to show a remarkable decline in the proliferation of tumors in the cancer-bearing mice. Thus, this system could prove to be a favorable approach for the effective treatment of tumors along with decreasing the frequent dosing regimen [66]. The Methotrexate, Platinum, Fluorouracil, Gemcitabine and CDK4/6 based chemotherapy and their combination polymeric nanoparticle drug delivery approach for breast cancer treatment are summarized in Table 4.

### 4.9. Vitamins Are Used for the Treatment of Breast Cancer through Polymeric Nanoparticles Drug Delivery System

#### 4.9.1. Vitamin E-Oligo (Methyl Diglycol l-Glutamate) and Tamoxifen

The clinical application of the Poly(d,l-lactide-co-glycolide) (PLGA) nanoparticles is limited due to the deficiency of biodegradable, biocompatible functional surfactants. Thus, this study aimed to formulate a bio-degradable, biocompatible type of surfactant using an amphiphilic vita-min E-oligo (methyl di-glycol l-glutamate) (VEOEG) to obtain tumor-specific nanomedicines. The paclitaxel-loaded HA-PLGA NPs (PTX-HA-PLGA NPs) were able to exhibit safer antitumor effects as compared to that of Taxol. At the same time, the in vivo pharmacokinetic study was performed on nude mice that displayed that the PTX-HA-PLGA NPs showed a better and longer plasma half-life of about 3.14 h. The in vivo biodistribution data showed that the PTX-HA-PLGA NPs displayed a higher level of tumor PTX of about 8.4% ID/g that was approx. 6 times higher and better as compared to that of Taxol. Consequently, PTX-HA-PLGA NPs displayed a better and safer inhibition in the proliferation of tumors and thus displayed fewer adverse effects and a better survival rate as compared to that of Taxol [67].

#### 4.9.2. Calcitriol

The clinical use of Calcitriol (1,25-dihydroxy vitamin D3) as an anti-cancer drug is restricted due to its drug-related hypercalcemia as well as the necessity of supraphysiological doses. Thus, calcitriol was encapsulated in a polymeric (PLA) nano-based formulation to limit drug-related problems. The in vitro studies were performed on MCF-7 breast cancer cell lines to assess the cytotoxic and anti-proliferative performance of the calcitriol. The study displayed that calcitriol was able to stimulate the anti-proliferative effects for 10 days as compared to that of free drugs in the cancerous cells. The reason for the anti-proliferative effect of the calcitriol was attributed to its nanoencapsulation efficiency that enhanced the drug retaining time at the tumor site [68].

### 4.10. Hormonal-Based Targeted Drug Delivery for Breast Cancer Using Polymeric Nanoparticles Approaches

#### 4.10.1. Trastuzumab and Docetaxel

In this study, Docetaxel-loaded D-α-tocopherol polyethylene glycol 1000 succinate conjugated chitosan (TPGS-g-chitosan) nanoparticles were synthesized for the treatment of breast cancer with or without Trastuzumab. The in vitro study was performed on SK-BR-3 cells that displayed that the docetaxel-loaded non-targeted and HER-2 receptor-targeted TPGS-g-chitosan nanoparticles were seen to increase the cytotoxicity and cellular uptake via a better bioadhesion property as compared to that of free docetaxel (Docel™). The in vivo pharmacokinetic study revealed that the bioavailability of docetaxel concerning non-targeted and HER-2 receptor-targeted nanoparticles was found to be 2.33 and 2.82 folds greater, respectively, as compared to that of Docel™. After the intravenous administration, the prolonged half-life was found to be 3.48 and 5.94-times greater as compared to that of Docel™ [69].

#### 4.10.2. Trastuzumab and Doxorubicin

The study aimed to prepare, characterize, and biological assessment of redox responsive polymeric nanoparticles for the effective delivery of doxorubicin for the treatment of breast cancer. In this study, folic acid was linked with the hydroxyl group via a technique called DCC-NHS coupling. The in vitro drug release study confirmed that the redox behavior of the nanoparticles displayed an approx. ∼72% release of doxorubicin in the presence of 10 mM GSH at pH 5.5 when compared to the in vitro drug release profile at pH 7.4. At the same time, the in vitro cellular uptake data manifested an average of ∼22% amount of cellular uptake in the presence of folic acid and trastuzumab linked nanoparticles made of a polymer when compared to that of non-targeted/non-specific nanoparticles. Whereas the fluorescence-activated cell sorting (FACS) study was performed on CF-7 cell lines that showed a high apoptosis percentage (∼80%) against the non-conjugated polymeric nanoparticles with only an apoptosis percentage of about ∼80%. The in vivo study showed a tumor regression percentage of about 91% in Ehrlich ascites tumor (EAT) in comparison with that of free doxorubicin administered mice [70].

#### 4.10.3. Bortezomib

The aim of the study performed was to synthesize and characterize a nanoformulation of folic acid- glycine-poly-L-lactic acid (FA-Gly4-PLA). The formulation was prepared to enhance the effectiveness and solubility of Bortezomib (BTZ), in which the BTZ encapsulated FA-Gly4-PLA was also formulated. The ROS intracellular activity was found to be higher in BTZ encapsulated FA-Gly4-PLA NPs. The apoptotic index was also found to be higher in BTZ loaded FA-Gly4-PLA NPs as compared to that of BTZ that was performed on MDA-MB-231 breast cancer cell lines. Thus, it was concluded that the BTZ encapsulated FA-Gly4-PLA-NPs were found to be more effective than BTZ loaded Gly4-PLA NPs as the FA-Gly4-PLA-NPs were able to load a much higher amount of BTZ [71].

#### 4.10.4. Exemestane

The present study aimed to synthesize and assess the exemestane (EXM) loaded TPGS-poly-ε-caprolactone based polymeric nanoparticles for increasing the antineoplastic activity. The in vitro cytotoxicity study showed that the cancerous cells were able to increase the cytotoxic activity possessed by EXM by the loading of TPGS-PCLNPs that was performed on the MCF-7 breast cancer cell line. It was observed that with the enhanced concentration of EXM in TPGS-PCLNPs, a sudden decrease in the cell viability was observed that displayed that the EXM-PLCNPs was able to show its better cytotoxic effects at various concentrations when compared to that of pure EXM. Whereas the reason for the increased cytotoxic effect was attributed to the nanosized formulation of EXM-TPGS-PCLNPs. Further, the study was concluded that the EXM-TPGS-PCLNPs could prove to be a better approach for the treatment of breast cancer as it would help to achieve better patient compliance in decreasing the dose-related adverse effects, dosing frequency [72].

#### 4.10.5. Herceptin

The study aimed to prepare docetaxel (DTX)-loaded poly(lactic-co-glycolic acid) nanoparticles (PNPs) with Herceptin^®^ (HCT) to enhance the cytotoxicity and drug internalization in the breast cancer cells. The poly(ethylene-alt-maleic anhydride (PEMA), surfactants, and numerous carboxyl groups were used for the preparation of PNPs. The cellular uptake study was performed for 2 h of HCT-N-PNPs and it was found to be 5.0-, 4.4, and 4.6-, 5.0-fold greater as compared to the PNPs in SK-BR-3, MCF-7 cells, BT-474 cells correspondingly. Whereas, when administered with at 40 μg/mL, the HCT-B-DTX- PNPs displayed a greater amount of toxicity concerning the SK-BR-3, MCF-7, and BT-474 cells against any other formulation. The study was concluded that the HCT-B-DTX-PNPs proved to be a better therapeutic approach in terms of possessing a higher amount of specificity and affinity and stimulating comprehensive cytotoxicity [73].

#### 4.10.6. Anastrozole

The study was aimed to prepare and evaluate anastrozole encapsulated various biodegradable polymeric nanoparticles viz, poly(ε-caprolactone) (PCL), poly(lactide-co-glycolide) (PLGA) and poly(lactic acid) (PLA). The polymeric nanoparticles were prepared using a simple emulsion technique. The cell viability assay for PLGA nanoparticles showed a comprehensive decline in the viability of the cell at different time intervals as compared to PCL and PLA nanoparticles. Whereas the AUC of PLC, PLGA, and PLA nanoparticles was found to be 19.81, 4.77, and 19.31-folds greater than free anastrozole solution [74].

#### 4.10.7. Letrozole

The study focused on designing, characterizing, and reducing the medicament-related adverse effects of the formulation. The polymeric nanoparticles (NPs) loaded medicament was designed for the effective treatment of hormone receptor-positive breast cancer. The medicament viz, Letrozole (LTZ) was loaded in poly(d,l-lactide) (PDLLA) nanoparticles (NPs) by the use of various concentrations of the LTZ with the help of emulsion solvent evaporation method. Whereas the amorphous nature of the LTZ was credited to the lower entrapment efficiency that led to the higher solubility of the drug in the polymer-based matrices that caused the transformation of the LTZ into amorphous nature. The in vitro study displayed that the LTZ showed enhanced release of the LTZ [75].

### 4.11. Curcumin and Their Combinational Approaches for Breast Cancer Treatment through Polymeric Nanoparticles Drug Delivery System

#### 4.11.1. Curcumin (Polyvinylpyrrolidone)

The study aimed to design nanoformulation consisting of folate-curcumin encapsulated gold- polyvinylpyrrolidone nanoparticles (FA-Cur Au-PVP NPs) to attain specific and targeted effectiveness of drugs for breast cancer treatment. The orthotropic mouse model was used for the assessment of the antitumor activity of the NPs. No accumulation of drug was seen when the NPs were incubated with human serum but was seen to mimic an internal peroxidase property when given with the substrate of 3,3′,5,5′-tetramethylbenzidine substrate. MTT assay displayed proficient antitumor efficacy even at lower doses with NPs when tested in the progesterone/estrogen (PR/ER) negative cells. Moreover, in an in vivo assessment, the NPs were able to display the antitumor and inhibitory effect [76].

#### 4.11.2. Curcumin (Chitosan)

The study aimed to prepare and fabricate curcumin encapsulated nanoparticles via a self-assembling technique. The targeting ability of the formulation was achieved by conjugating it with folate and thus preparing the curcumin encapsulated folate-engineered chitosan-nanoparticles (NPs). The in vitro release study showed an enhancement in the release rate of curcumin from the NPs that was performed on MCF-7 breast cancer cell lines. While the cell viability studies proved that curcumin encapsulated NPs can be used as an effective approach for the treatment of breast cancer [77].

#### 4.11.3. Curcumin (Alginate/Chitosan)

In this study, curcumin diethyl disuccinate (CDD) was synthesized using alginate/chitosan by the use of Box-Behnken statistical design to augment the therapeutic efficiency of curcumin diethyl disuccinate. The CDD encapsulated alginate/chitosan nanoparticles were prepared by an oil-in-water emulsification technique that was succeeded by an ionotropic gelification. The in vitro cytotoxicity study revealed that the CDD showed a better and safer stability profile with an enhanced cytotoxic effect when compared to curcumin. The cellular uptake studies that were performed on MDA- MB-231 cells were seen to be higher too when compared to that of free CDD. Moreover, the chemical and physical stability studies showed that the formulation of CDD encapsulated alginate/chitosan nanoparticles was found to be stable at 4 °C for 3 months [78].

#### 4.11.4. Curcumin and Methotrexate

In a study, methotrexate (MTX) and curcumin (CUR) loaded PLGA nanoparticles were synthesized and characterized for various parameters for the treatment of breast cancer. The in-vitro¬ release profile of MTX-CUR loaded nanoparticles displayed a rapid release as compared to that of free MTX and CUR solutions. A comparison study was done between MTX-CUR nanoparticles, free MTX solution, and CUR solutions that dis-played that MTX-CUR nanoparticles displayed a comprehensive greater amount of cytotoxicity than that of free MTX and CUR solutions. The in vivo study displayed that the dual therapy of MTX and CUR loaded nanoparticles led to the development of synergistic activity possessed by both the drugs and thus caused a decline in the proliferation of tumors [79].

#### 4.11.5. Curcumin and Gemcitabine

In a study, curcumin (CUR) was loaded with gemcitabine (GEM) to attain a synergistic effect. One nanoemulsion of CUR/GEM has individually conjugated with folate and the other nanoemulsion of the dual drug-loaded viz, CUR+GEM, was conjugated with folate ligand. The nanoemulsions were further evaluated for scintigraphic imaging and P-glycoprotein-1 (Pgy-1) gene resistance and it was observed that the formulations that were conjugated with folate ligand (FCGNPs) displayed better effects in terms of Pgy-1 along with it, they displayed better and greater cellular apoptosis, cellular uptake, cellular cytotoxicity, and cell cycle arrest in a better manner. The FCGNPs displayed a greater deposition of drugs in the MDA-MB-231 breast cancer cell lines as compared to that of the non-folate conjugated formulations. The in vivo studies exhibited that FCGNPs were found to be safer, higher, and efficacious in terms of delivering therapeutic effect as compared to that of other formulations. Thus, it was concluded that dual therapy could prove to be a better approach for the treatment of breast cancer that works by delivering better cytotoxic profiles and thus leading to a decline in breast adenocarcinoma, thereby decreasing the resistance of the drug [80].

The vitamins, hormones, and phytoconstituents and their combination polymeric nanoparticle drug delivery approach for breast cancer treatment are summarized in Table 5.

### 4.12. Miscellaneous Examples of the Drug Used for Breast Cancer Treatment Using Polymeric Nanoparticles Drug Delivery System

#### 4.12.1. Nimbolide (Nim)

The present study aimed to formulate nanoparticles of Nimbolide (Nim-Nano) using poly(lactic-co-glycolic acid) (PLGA) to enhance the therapeutic effectiveness of the hydrophobic drug. The Nim-Nano was synthesized with the help of a technique called nanoprecipitation and was further characterized. The assessment study of the anticancer effect of Nim-Nano was performed on pancreatic AsPC-1 and MCF-7 and MDA-MB-231 cancer cell lines that displayed the Nim-Nano showed a continuous release pattern of Nim for 6 days and more. In addition, it displayed a 2–3 folds increase in the cytotoxic effect in pancreatic and breast cancer cell lines as compared to that of free Nim [81].

#### 4.12.2. Disulfiram

In a study, poly (lactide-co-Glycolide) (PLGA)-Polyethylene glycol (PEG) nanoparticles were prepared that were self-engineered using a folate ligand. The folate-receptor-targeted nanoparticles were prepared for the effective delivery of disulfiram in the cancerous breast cells. The folate-receptor-targeted nanoparticles hindered the growth of cancerous cells, led to the induction of apoptosis, and also caused internalization of disulfiram into the cancerous cells in a better way as compared to that of the non-targeted nanoparticles. The toxicity study showed that the highest dose of disulfiram via intravenous route was 6 mg/kg, which displayed a remarkable decline in the tumor of the breast cancer cells [82].

#### 4.12.3. Suramin (SM) and Doxorubicin

In a study, suramin (SM) and glycol chitosan (GCS) nanogels were loaded with doxorubicin (DOX) nanoparticles (GCS-SM/DOX-NPs) were designed and assessed for their efficacy in breast cancer treatment. The in vitro study displayed that the GCM-SM nanoparticles were able to induce angiogenesis and were able to hinder the migration of the cancerous cells. The DOX NPs/GCS-SM led to an increase in the lifespan of animals by decreasing the proliferation of tumors in a TNBC model of lung metastasis, thereby reversing the adverse effects of the DOX and SM consequently. Thus, it was concluded that GCS-SM/DOX-NPs could be used as an effective therapeutic tool for the treatment of metastatic breast cancer as it not only stimulates angiogenesis, thereby lowering the drug-related adverse effects but because of the presence of the biocompatible products used in them that makes them easy for the production of largescale formulation products [83].

#### 4.12.4. Nimesulide (NMS)

The current study aimed to design poly(ethylene glycol)-block-poly(ε-caprolactone) (PEG-b-PCL) nanoparticles loaded with nimesulide (NMS) that were characterized to assess its anticancer property on MCF-7 breast cancer cells. PEG-b-PCL loaded NMS nanoparticles exhibited globular shape. The PEG-b-PCL loaded NMS nanoparticles displayed its anticancer effect in a dose-dependent way in MCF-7 breast cancer cell lines. It was observed that the PEG-b-PCL loaded NMS nanoparticle’s influence was greatly dependent on the method of preparation of the nanoparticles. The study concluded that the PEG-b-PCL loaded NMS nanoparticles displayed a proficient result due to its nanosize and presence of NMS that led to pro-apoptotic effect in the MCF-7 breast cancer cell lines, therefore in the future, this approach could be aided as a better and effective approach for the treatment of breast cancer [84].

#### 4.12.5. Piceatannol

The study aimed to design poly-lactic acid (PLA) and chitosan (CS) encapsulated nanoparticles (NPs) for the delivery of piceatannol (PIC). The NPs were seen to display excellent scavenging properties for NO, SOD, and DPPH radicals. Whereas the LDH and MTT assays displayed that the greater and effective cytotoxic effects of CS/PLA-PIC NPs in MCF-7, A549, and HepG2 cells when compared to that of CS-PLA-PIC and CS-PLA-NPs. Whereas the results of the double staining exhibited various late/early phases of the necrotic and apoptotic cells. Additionally, the results of flow cytometry displayed strong proliferative activity in human breast, liver, and lung cancer cell lines by CS/PLA-PIC NPs and thus, it was concluded that CS/PLA-PIC NPs led to the variation in the protein expression of apoptosis, thus stimulating the mitochondrial-dependent apoptosis in the cancerous cells and tissues [85].

#### 4.12.6. Artemisinin

The study aimed to design magnetic nanoparticles (NPs) of artemisinin (AR) with the help of the ionic gelation technique using gelatin. The prepared AR-NPs displayed a rapid release of about 62%–78% for about 48 h. In 4T1 breast tumor tissues of BALB/c mice model, the FTIC linked AR-NPs displayed an enhanced deposition of NPs at the site of the tumor. Thus, it was concluded that the formulation could be a better approach for breast cancer treatment [86].

#### 4.12.7. Rapamycin and Piperine

The current study aimed to design and evaluate Rapamycin’s RPM) nanoparticles using a polymer named poly(d,l-lactide-co-glycolide) (PLGA) and a chemosensitizing agent viz, piperine (PIP) to enhance the effectiveness and the oral bioavailability of the formulation. The effects of p-glycoprotein (P-gp) were evaluated using an everted gut-sac technique that confirmed that the uptake of the RPM was enhanced because of the aid of PIP, while the pharmacokinetic study displayed that the NPs exhibited a better absorption property by 4.8 folds as compared to that of RPM’s suspension. Thus, it was concluded that the dual approach of RPM and PIP-loaded NPs would prove to be a better tool for breast cancer treatment [87].

#### 4.12.8. Honokiol

The study aimed to formulate honokiol (HK) loaded in PEGylated-PLGA polymeric nanocapsules (NCs) for the treatment of breast cancer. The in vitro studies performed in EAC and MCF-7 breast cancer cell lines, while the in vivo studies performed with the help of solid Ehrlich carcinoma (SEC) indicated the anti-tumor efficacy of the HK-loaded NCs. The HK-loaded NCs were seen to hinder the proliferation of cancerous cells in an in vitro study with approximately 80.2% and 58.1% as compared to that when treated with free HK solution in EAC and MCF-7 breast cancer cell lines that were found to be 31% and 35%, respectively. The study was concluded, and it was found that the HK-loaded NCs blocked the proliferation of tumor (SEC) by 2.3 folds that remarkably greater than that of free HK that proved the effectiveness of the novel formulation of HK-loaded NCs in the treatment of breast cancer [88].

#### 4.12.9. Ormeloxifene

The current study aimed to design Ormeloxifene encapsulated PEGylated chitosan nanoparticles (CNPs) to enhance its anticancer activity for the treatment of breast cancer. The CNPs, as compared to that of free ormeloxifene against the MCF-7 and MDA-MB-231 breast cancer cell lines, were able to display an increased cellular uptake, apoptosis, stimulation of caspase-3, destruction of the membrane of the mitochondria, cytotoxicity in a dose-dependent way. The in vivo studies demonstrated an enhanced rate of survival, the diminished burden of the tumor, and diminished adverse effects in female Sprague-Dawley rats treated with CNPs. Thus, it was concluded that the CNPs approach could prove to be a better approach for the treatment of breast cancer [89].

#### 4.12.10. Psoralen

The present study aimed to design psoralen polymer-lipid hybrid nano-particles (PSO-PLN) to converse the drug-resistant MF-7/ADR cells. The in vitro data indicated that the PSO-PLN resistance index was found to be 5.6, whereas the PSO resistance index was about 3.2, that the reversal effect of PSO-PLN against MCF-7/ADR was found to be significant. Furthermore, the PSO-PLN was also able to display its antitumor efficacy in a tumor xenograft model. Thus, the study concluded that the PSO-PLN converses the rate of MDR, while the therapeutic effectiveness of the PSO-PLN was attributed to the increment in the continuous release of the PSO [90].

#### 4.12.11. Niclosamide

The study aimed to design nanoparticles (NPs) of niclosamide (NIC) and silver (Ag) in a nanofiber-based system. The anticancer effects of free NIC/Ag alone and in a nanofiber combination viz, NIC- Ag-NPs was assessed using MTT assay technique that showed a proficient and effective delivery of the NIC and Ag-based nanofibers as compared to that of free NIC/Ag against MCF-7 (breast carcinoma) and A549 (lung carcinoma) cell lines. Besides, NIC-Ag-NPs displayed improved therapeutic effectiveness in MCF-7 breast cancer cell lines. Thus, it was concluded that the NIC-Ag-NPs could be used as a better carrier for breast cancer treatment [91].

#### 4.12.12. Etoposide and Quercetin

In a study, the etoposide’s bioavailability was increased via co-loading it with quercetin nanoparticle formulation (EQNP). The cytotoxicity study showed 11 folds increase in the IC50 value of the EQNP as compared to that of ENP that showed only a 9-folds increase in the IC50 value. The confocal imaging study of the intestinal portions, when treated with EQNP and ENP, displayed that the EQNP was able to permeate deeper in the intestine. Moreover, the pharmacokinetic study showed EQNP displayed 2.4-folds enhancement in the etoposide’s bioavailability as compared to that of ENP. Thus, the prepared formulation of EQNP proved to be a safer and effective approach to minimize the drug-associated adverse effects, sensitize the resistant cancerous cells and enhance the bioavailability of etoposide [92].

#### 4.12.13. Evodiamine

The current study aimed to enhance the bioavailability and increase the solubility of evodiamine (EVO) via encapsulating EVO with poly (lac-tic-co-glycolic acid) (PLGA) nanoparticles EVO (EVO-PLGA NPs) to treat breast cancer. The release of EVO-PLGA NPs could control the release of EVO for 180 h. The EVO-PLGA NPs formulation was able to down-regulate the β-actin expression and upregulate the cyclin B1 expression that proving its effectiveness for the treatment of breast cancer therapy [93].

#### 4.12.14. Sclareol

The study aimed to load sclareol in PLGA nanoparticles (NPs) to enhance its anticancer property. It was seen that sclareol did not show any physical destabilization. The coating of the PLGA-NPs showed an enhanced anti-tumor effect of the loaded sclareol in MCF-7 and MDA-MB468 human breast cancer cells and CaCo-2 human colon carcinoma cells [94].

The various chemotherapy and phytoconstituents and their combination polymeric nanoparticle drug delivery approach for breast cancer treatment are summarized in Table 6.

## 5. Limitations and Challenges of Polymeric Nanoparticles

The major limitations and challenges of polymeric nanoparticles are to avoid the systemic toxicity and inflammation of the polymer used because of the accumulation of these polymer-based nanoparticles in the tissue till the toxic level, which is caused due to the hindrance of getting degraded or excreted from the body. So, to overcome this limitation, biodegradable polymers are widely used by research groups in the development of polymeric nanoparticles to reduce toxicity, improve drug release and increase biocompatibility, for example, polylactic acid polylactic glycolic acid, and polycaprolactone, etc. In addition, a few natural polymers, including alginate, chitosan, albumin, and gelatin, have also been used in the development of polymeric nanoparticles [95]. Another limitation is the attachment of ligand molecules, e.g., folic acid, transferrin, estrone, etc., to the surface of a polymer through conjugation for better drug targeting to avoid the unwanted peripheral side effect or systemic toxicity. As in breast cancer, the overexpression of various receptors is very high, which are clinically used to target the breast cancer cells for effective drug delivery by conjugating the ligand molecules as mentioned above to the drug-coated polymer. The difficulty faced in conjugating the ligand to the polymeric nanoparticles is due to the surface complexity of polymer and conjugation reaction accuracy. Hence, this leads to a very difficult stage for their evaluation and scale-up for formulation development and again, a major concern is the reproducibility parameter which makes a very cost-effective process [96,97].

## 6. USFDA Approved Polymeric Nanoparticles

Polymer-based nanoparticles of various medicines approved by USFDA in the various timeline has been mentioned in Table 7.

## 7. Conclusions and Recommendation

The present review summarized the various chemotherapeutic drugs and their combination formulated in polymeric nanoparticles for the promising and effective drug delivery for breast cancer treatment. The chemotherapy-related drugs have their properties which make them the more therapeutic value of less depending upon the factors associated. Sometimes the properties of the drug may compromise and overcome the properties to gain maximum benefits in respect of therapeutic values they formulated into nanotechnology drug delivery approaches, which could be better as compared to conventional therapy. Polymeric nanoparticles drug delivery systems have been summarized in this review manuscript for promising and effective drug delivery in breast cancer treatment. The result of this approach summarized which indicated the benefits over the inherent lacuna of chemotherapeutics drugs which are discussed in each section of this review manuscript.

With an increase in the number of cases each year, breast cancer is known to be the causative factor for the second foremost death in women worldwide. The available first-line treatment includes the use of a chemotherapeutic approach such as radiation, surgery, and hormonal therapy, due to some hurdles like various side effects such as multi-drug resistance and high hepatic metabolism that delivers a less effective dose of chemotherapy to the target site. The nanotechnological approach like polymeric nanoparticles for drug delivery is an alternative pathway for effective dose delivery and also to overcome the issues related to therapeutic efficacy. The polymeric nanoparticles type of drug delivery is still an underdeveloped formulation as few marketed formulations are available such as (Abraxane) for breast cancer treatment.

As per the data related to the marketed or clinical trials, the formulations that would create a broad white space in this area of research are not much available. So, the area of research that focuses on the in vitro as well as the in vivo result outcome could provide the precise data during the scale-up of the same when applied with big scale production. Apart from scale-up, toxicological assessment much-needed parameter for polymeric nanoparticles, for which there is insufficient literature available make a difficult choice of drug delivery system. Nowadays, the area of focusing research is also working on the dual drug delivery system in which co-encapsulated drugs polymeric drug delivery system. Based on the combinational index, we can expect the synergistic effect of the combination drug approach could overcome such therapeutic barriers.

The authors also included highly investigated chemotherapeutics such as paclitaxel, docetaxel, and doxorubicin. Apart from chemotherapy, curcumin was investigated widely in the phytoconstituents category. The data also suggests that this research is only focused on these 3–4 chemotherapeutics drugs and not on the newly approved drugs by FDA or recent ones. Breast cancer is a promising area of research work in which various approaches of drug delivery are possible for academic research work, including the polymeric drug delivery, approaches. It would be better if the research work focusses on recently approved drugs of conventional drug delivery to give a better outcome.

## Figures and Tables

**Table 1:** Paclitaxel and their combination polymeric nanoparticles for breast cancer treatment.

DRUGS	LACUNA ASSOCIATED	POLYMER USED	METHOD OF PREPARATION	IN VIVO/CELL LINE STUDY	OUTCOME	REFERENCES
PTX	Poor water solubility Poor bioavailability Poor penetrating ability of PTX	Alpha-tocopheryl polyethylene glycol 1000 succinate-block-poly (caprolactone) and methoxy-PEG block-poly (caprolactone)	PEGylating strategy	MCF-7 and MDA-MB-231	Improved cytotoxic effect Enhanced anti-tumor effect Upgradation in the cellular uptake efficacy	[11]
PTX	Hydrophobicity High hemolytic toxicity	Chitosan	Water in oil nanoemulsion method	MDA-MB-231	4 folds reduction in the hemolytic effect of the NPs loaded with PTX on comparison with other formulations Improvement in the % of apoptotic cells by the formulation 2-times higher apoptotic effect by the formulation	[12]
PTX	Drug resistance Poor water solubility	Chitosan and poly (di (ethylene glycol) methyl ether methacrylate) (PDEGMA)	Grafting followed by dialysis	MDA-MB-231 and Human umbilical vein endothelial (HUVEC)	Insignificant cytotoxicity in HUVEC cell and higher toxicity in cancerous MDA-MB-231 cell line by HA-CS-g-PDEGMA-PTX NPs Extensive range of apoptosis of tumor cells was observed by PTX loaded NPs than free PTX	[13]
PTX	Multiple drug resistance Dose-related side effect Low targeting efficiency	PLGA	Solid-in-o/w evaporation method, trailed by Tf adsorption on the surface	MCF-7 and U-87	Improved cytotoxicity effect and cellular efficiency under the influence of Tf receptor due to the endocytosis Upgradation of the cellular uptake efficiency in the presence of magnetic field Enhanced bioavailability of the PTX	[14]
PTX	Low aqueous solubility Side effects associated with a higher dose of PTX such as hypersensitivity, hypotension and neurotoxic effect	Keratin (KER)	Simple and straightforward aggregation method	MCF-7 and MDA MB 231	Significant decrease in the % of living cells followed by a significant increase in early apoptosis by the PTX-KER-NPs Improved penetrability of the PTX in the cancerous cells Enhanced anti-tumor effect Improved targeting efficiency further led to the enhancement in the bioavailability	[15]
PTX, GNPC and ES	Poor targeting capability of the drug Poor solubility Poor penetrating ability	Chitosan	Graft copolymerization	MCF-7 and MCF-10A cell lines	Penetration of PTX-GNPC-ES was 5 folds higher than GNPC in the cells %Tumor inhibition rate was found in order of PTX-GNPC-ES > PTX-GNPC > PTX solution	[16]
PTX	Drug resistance towards the triple-negative mammary cancer	PLGA coated with HA	Modified oil-in-water emulsion method	MDA-MB-231 cells	no hemolytic toxicity observed enhancement in the cellular uptake of PTX	[17]
PTX and SLM	Low solubility Non-specificity in the distribution of the drug Unsought biopharmaceutical properties	PLGA	Emulsion solvent diffusion method followed by cationic stabilizers	MCF-7 and MDA-MB-231 cells	Synergistic effect was observed in the cytotoxicity activity to the % growth of CD44^+^ cells Upgradation in the bioavailability of the drugs Prolonged circulation time of the drugs	[19]
PTX and GEM	Multiple drug resistance Drug-related toxicity	MPEG-PLA (methoxy poly (ethylene glycol)-poly (lactide-coglycolide)	Film hydration method	4T1, MCF-7, and MDA-MB-231	improved anti-cancerous effect low systemic toxicity enhanced targeting efficiency	[20]
PTX and FA	Poor targeting efficiency Lower penetrating ability	poly(ε-caprolactone) (PCL), PEG, DSPE-PEG2000 (1,2-distearoyl-sn-glycero-3-phosphoethanolamine-N- [methoxy (polyethylene glycol)-2000])	Thin-film hydration and ultrasonic dispersion method	EMT6 cell lines	Cytotoxic effect was observed in order (Taxol^®^ > PTX-FLNPs > PTX-LNPs (without FA) 1.3 folds higher inhibition of the tumor growth was discovered by PTX-FLNPs than PTX-LNPs	[21]

**Table 2:** Docetaxel and their combination polymeric nanoparticles for breast cancer treatment.

DRUGS	LACUNA ASSOCIATED	POLYMER USED	METHOD OF PREPARATION	IN VIVO/CELL LINE STUDY	OUTCOME	REFERENCES
DTX	Poor targetability of the drug Poor penetrability of the fluorescence	Amphiphilic theragnostic fluorescence labelled polymer	O/W emulsion following solvent evaporation method with few alterations	MDA-MB-231	Effective drug loading Upsurged cytotoxicity with 3 to 4.4 folds reduction in the dose of a drug required for IC_50_ value Increase MRI contrast	[22]
DTX	Poor water solubility Multiple drug resistance	PAMAM-based poly (γ-benzyl-l-glutamate)-b-d-α-tocopheryl polyethylene glycol 1000 succinate (PAM-PBLG-b-TPGS)	Nanoprecipitation method	Hela cells and MCF-7	Stronger cytotoxic effect Improved cellular uptake efficacy Potent anti-tumor effects Improved delivery of DTX at the target site	[23]
DTX	Multiple drug resistance P-gp efflux of DTX Severe side effects associated with Poloxamer 235	PLGA/TPGS	Modified Nanoprecipitation method	MCF-7/TXT	Increased in the level of DTX uptake from PLGA-TPGS/Poloxamer 235 NPs compared to PLGA-TPGS NPs Higher level of cytotoxicity was observed with Poloxamer conjugated NPs Surpassed MDR effect Enhanced concentration of DTX in the cells	[24]
DTX	Taxane-resistant triple-negative breast cancer Dose-limiting toxicity	PLGA	An imprint lithography-based technique denoted as Particle Replication in Nonwetting Templates (PRINT)	C3(1)-T-antigen (C3Tag) genetically engineered mouse model (GEMM) of breast cancer	DTX loaded NPs showed 376 folds increase in the plasma concentration of the DTX over-marketed formulation 2 times enhancement in the DTX accumulation in the tumor	[25]
DTX	Resistance to chemotherapy Dose-dependent side effects	PLGA conjugated with FA	solvent evaporation method	Human breast cancer cells	Markedly reduced the expression of ABCG2 by 3.2-folds and MDR1 by 2.86-fold High apoptotic effect	[26]
DTX	Toxic effect and side effects associated with higher dose on the normal cells	PHBV	Modified emulsification solvent evaporation method	MCF-7	Enhanced inhibition by the NPs followed by advanced apoptotic effect	[27]
DTX	Harmful side-effects related to the non-specificity of the drug distribution Poor aqueous solubility Susceptibility to the P-gp efflux pump	PEG-PLGA functionalized by E2	Emulsion diffusion evaporation method	Hela cells and MCF-7	12.90 times increase in the circulation half-time of the drug Increase permeability and retention time of the drug Improvement in the cellular uptake efficacy of the DTX	[28]
DTX	Low aqueous solubility Intense phagocytic effect Rapid clearance Non-specific nature	Chitosan and sodium tripolyphosphate (TPP) act as a cross-linkers.	Ionotropic gelation method	MDA-MB-231	85% observed reduction in the cell viability Enhancement in the targeting approach due to cross-linker	[28]
DTX	Resistance and Side effects of Herceptin Ligand density	PLGA	Modified emulsification technique	SK-BR-3	Better therapeutic efficacy Reduction in then burst release followed by sustained release Advanced synergistic effects	[29]
DTX and THQ	Poor aqueous solubility Prone to P-gp effluxAssociated side effects with DTX such as hypersensitivity, hypertension, etc.	Chitosan	High-speed homogenization and ultra-sonication	MDA-MB-231 and MCF-7	substantially advanced cytotoxicity contrary to triple-negative Improved anti-angiogenic effect Increased uptake in addition to endosomal escape effect Advanced anti-angiogenic effects	[30]
DTX and SFN	Rapid clearance of the drugs Non-specificity towards the organ	PLGA and HA	The method of preparation has been described by Jeong and associates	Differentiated breast cancer cells (DBCCs) and Breast cancer stem cells (BCSCs)	Subdued the self-activating ability of BCSCs Strong anti-tumor activity of the combination Synergistic effects	[31]
DTX and GEM	Non-specific accumulation of the drug Unrestrained pharmacokinetic profile of DTX and GEM individually Speedy metabolism of GEM	PEG	Followed general procedure for the development of the NPs	MCF-7 and MDA-MB-231	Upsurged cellular uptake of NPs via clathrin facilitated endocytosis. Higher inhibition in the tumor growth Less toxicity was observed in the liver and kidney on treatment 4.8-fold higher AUC _(0–∞)_ of GEM compared to the marketed formulation	[32]
DTX and SAL	Low solubility Low oral bioavailability	PLGA/TPGS(Tocopheryl polyethylene glycol 1000 succinate)	Nanoprecipitation method	MCF-7 and MCF-7-MS	Synergistic effect Improved cytotoxic effect Extended circulating time Enhanced targeting efficiency	[33]
DTX and Hyaluronic acid	Low targeting efficacy Poor solubility Dose-dependent toxicity Allergic reactions	Chitosan and Hyaluronic acid	Spontaneous ionotropic gelation method	MCF-7	Reduction in the cell viability Significant decrease in gene expressions in the cells	[34]

**Table 3:** Doxorubicin and their combination polymeric nanoparticles for breast cancer treatment.

DRUGS	LACUNA ASSOCIATED	POLYMER USED	METHOD OF PREPARATION	IN VIVO/CELL LINE STUDY	OUTCOME	REFERENCES
Doxorubicin	Off-target toxicity	PLGA-PEG-COOH and PLA-PEG-OCH3	Nanoprecipitation method	MDA-MB-231	Reduction in cell viability High growth inhibition Cytotoxic, cytostatic, and/or antiadhesive effects	[35]
Doxorubicin	Lack of ability to target precisely Limited their effectiveness	mPEG-PLGA	Nanoprecipitation method	MDA-MB-831	High preferential cytotoxicity Extended circulation and retention	[36]
Doxorubicin	Adverse toxicities related to drug	PEG	Seed mediated method using surfactant	MCF-7 and MDA-MB-231	Overexpression of pro-apoptotic protein p53 Decrease in receptors of the anti-apoptotic protein Bcl-2	[37]
Doxorubicin	Lack of specificity Low solubility Rapid elimination Non-specific distribution Dose-related toxicities	PLGA and DSPE-PEG 2000	Modified nanoprecipitation	MDA-MB231 and PC3	Good biocompatibility Higher degree of particle internalization Higher antiproliferation effects	[38]
Doxorubicin	Off-target	Poly (N-vinylcaprolactam) chitosan	Dialysis method	TNB xenograft mouse model	Less off-target cytotoxicity Reduction in tumor volume No obvious systemic toxicity	[39]
Doxorubicin	Low solubility Side effects	O-succinyl chitosan and Pluronic^®^	Self-assembly method	MCF-7 and Vero cells	Increase in the therapeutic efficacy Higher therapeutic efficacy Higher anticancer activity toward the HER2 overexpressing cancer cells	[40]
Doxorubicin and Indocyanine green	Drug resistance Reduced treatment efficacy	PCL and PEG	Thin-film hydration and ultrasonic dispersion	EMT-6	Enhanced reduction-sensitivity Synergistic cytotoxicity Improved the uptake of nanoparticles	[41]
Doxorubicin and Celecoxib	Multidrug resistance Reduce the intracellular accumulation	HPPDC	Emulsion-solvent evaporation method.	MCF-7/ADR	Tumor-targeting Enhanced chemosensitivity COX-2 and P-gp reduced expressions.	[42]
Doxorubicin and Curcumin	Drug resistance	mPEG-PLGA-PGlu	Nanoprecipitation method	MCF-7/ADR and Xenograft mice model	Enhance DOX distribution in tumor Useful strategy for refractory breast cancer	[43]
Doxorubicin and Curcumin	Fast clearance Unspecific distribution	PEG	Solvent evaporation	MCF-7 cells and mice bearing MCF-7 cells	Enhanced cytotoxicity Efficient tumor-targeted Exhibited stronger antitumor effect	[44]
Doxorubicin and Curcumin	Adverse effects related to drug	Sodium alginate hydroxyapatite	Simple diffusion deposition approach	MCF-7 and HEpG2	Inhibit the growth of MCF-7 (breast) and HEpG2 (liver) cells Non-toxic towards normal cells	[45]
Doxorubicin, 5-fluorouracil and Cisplatin	Poor water solubility	PCL-PEG	Double emulsion method	T47D and MCF7	Improve the stability and solubility Enhanced cytotoxicity	[46]
Doxorubicin Noscapine	Multi-drug resistance Adverse side effects	mPEG and PLGA	Nanoprecipitation method	4T1 and mice model	Synergistic anticancer effects Inhibited tumor growth Antiangiogenic effect	[47]
Doxorubicin and core Ag	Cardiotoxicity Cancer resistance Bone marrow suppression	PVA, PEG and PVP	Chemical reduction	MCF-7 and 1BR hTERT	More cytotoxic More effective cancer therapeutics Lower cytotoxic 1BR hTERT cells	[48]
Doxorubicin and Cisplatin with core Aldehyde Hyaluronic Acid (AHA)	Rapid development of multidrug resistance Systemic toxicity	Hydroxyethyl chitosan (HECS)	Schiff’s base bond and electrostatic interactions.	MCF-7	Enhanced the cellular uptake Synergistic cell-killing effect Synergistic combination	[49]
Doxorubicin Chlorin e6 and Manganese dioxide	Limitations of conventional chemo treatment	PCLA-PEG-PCLA	W/O/W emulsion solvent evaporation method	MCF-7 tumor-bearing mouse model	High stability and biocompatibility Decomposition of excessive endogenous H2O2	[50]
Doxorubicin and Pyrrolidinedithiocarbamate	Serious adverse effects MDR	Poly (ortho ester urethanes)	O/W emulsion solvent evaporation method	MCF-7 and MCF-7/ADR cells	Reverse MDR Enhanced intracellular drugs accumulation Expression of P-gp reduced Superior tumor growth inhibition	[51]
Doxorubicin and Quercetin	Multidrug resistance	PEG-PCL	Thin-film hydration method	MCF-7/ADR cells	Effect of doxorubicin resistance MCF-7/ADR breast cancer cells reduced Inhibition of both the activity and expression of P-glycoprotein	[52]
Doxorubicin and Disulfiram	Multidrug resistance Systemic toxicity	PCL-b-PGlu-g-mPEG	Dialysis method	MCF-7 and MDA-MB-231	Improved intracellular accumulation Synergistic cytotoxic effect	[53]
Doxorubicin and Metformin	P-glycoprotein (P-gp) MDR	PLGA and TPGS	Double emulsion method	MCF-7	Higher cytotoxicity and apoptosis Reduced drug efflux and increased cellular uptake Reducing cellular ATP content and inhibiting the effect of P-gp.	[54]

**Table 4:** Methotrexate, Platinum, Fluorouracil, Gemcitabine and CDK4/6 based chemotherapy and their combination polymeric nanoparticles for breast cancer treatment.

DRUGS	LACUNA ASSOCIATED	POLYMER USED	METHOD OF PREPARATION	IN VIVO/CELL LINE STUDY	OUTCOME	REFERENCES
Methotrexate	Poor biodistribution Stability issues	Chitosan	Biosynthesized	MCF-7 breast cancer cells	Destruction of the membrane in the cells of MCF-7 Upregulation of caspase3	[55]
Methotrexate	Poor solubility Stability issues	PLGA polymer	Double emulsion method	T47D breast cancer cells	Enhanced cytotoxic effect	[56]
Methotrexate and Aceclofenac	Major adverse effects of the drugs	Lipid polymer	Modified single step self-assembled nanoprecipitation method	MCF-7 and MDA-MB-231 cells of breast cancer	Antitumor efficacy Prophylactic effect Synergistic property	[57]
Carboplatin	Poor bioavailability Major side effects of the drug	Chitosan	Ionic interaction procedure	MCF-7 breast cancer cell line	NPs enhanced the safety, biodegradability, Enhanced the circulation time of drugs in blood, Increased the stability of drug Provided sustained release	[58]
Cisplatin	Drug associated adverse effects include kidney problems	Chitosan	Iron oxide method	MDA-MB-231 breast cancer cell lines	Enhanced the cytotoxic effect and apoptosis	[59]
Cisplatin	Drug associated adverse effects	Dextran	Complexation and dialysis method	MCF-7 and 4T1 cells	Enhanced cellular uptake and cytotoxicity Reduced systemic toxicity	[60]
5-Fluorouracil	Short half-life Expansion of the drug resistance criteria via cells of the tumor	PEG-PLGA	Nanoprecipitation solvent evaporation technique	Normal CCD-18, MCF-10A cells and tumour HT-29 and MCF-7 cells	Showed half-maximal inhibitory concentration	[61]
5-Fluorouracil and Taribavirin	Side effects associated with the drug	PEG	Modified emulsification and solvent evaporation method	MCF-7 breast cancer cell lines	Proficient cytotoxic effects Enhanced therapeutic index	[62]
Gemcitabine	Drug associated adverse effects	Fucoidan and Chitosan	Polyelectrolyte complexation	MDA-MB-231, EA.hy926, cell line	Enhanced toxicity of about 25% in comparison with free gemcitabine	[63]
Gemcitabine	Drug associated adverse effects	N-trimethyl chitosan	Ionic gelation	4T1 cell line	Enhanced bioavailability Decreased the proliferation of tumor	[64]
Dasatinib	Poor solubility Low therapeutic efficacy Adverse effects of drug	Polylactide, Poly(cyclohexene phthalate)	Nanoprecipitation	HEK-293 cell line and the MCF10A cell line	Targeted drug release	[65]
Lapatinib	Adverse effects linked with the drug	PLGA	Ring-opening polymerization technique	MCF-7	Decreased the proliferation of tumor	[66]

**Table 5:** Vitamins, Hormones, Phytoconstituents and their combination polymeric nanoparticles for breast cancer treatment.

DRUGS	LACUNA ASSOCIATED	POLYMER USED	METHOD OF PREPARATION	IN VIVO/CELL LINE STUDY	OUTCOME	REFERENCES
Vitamin E and Tamoxifen	Adverse effects linked to the drug	PLGA	Emulsion solvent evaporation technique and Nanoprecipitation method,	MCF-7 Cells, U87MG Cells, L929 Cells	Better survival rate ↓ Adverse effects and anticancer effect	[67]
Calcitriol	Adverse effects linked to the drug	Calcitriol	Nanoprecipitation technique	MCF-7	Anti-proliferative effect Drug retaining time at tumor site increases	[68]
Trastuzumab and Docetaxel	Adverse effects linked to the drug	Chitosan	Modified solvent evaporation method	SK-BR-3	Enhanced bioavailability Half-life of the drug-enhanced	[69]
Trastuzumab and Doxorubicin	Drug-related side effects	PLA-PEG-PLA	Nanoprecipitation method	MCF-7, BT 474, and L929 cell line	Enhanced cellular uptake Regression of tumor also increase	[70]
Bortezomib	Poor solubility	PLA	Modified solvent evaporation technique	MDA-MB-231	Enhanced apoptotic index Therapeutic effectiveness	[71]
Exemestane	Poor solubility Poor lipophilicity	TPGS	Nanoprecipitation method	MCF-7	Enhanced cell viability ↑ Cytotoxicity	[72]
Herceptin	Drug linked side effects	PEG	Emulsion-solvent evaporation method with modifications	SK-BR-3, MCF-7 cells, BT-474 cells	Enhanced the cytotoxicity Drug internalization in the breast ↑	[73]
Anastrozole	Poor solubility	PLGA, PLA and PCL	Simple emulsion technique	BT-549 and MCF-7	Enhanced circulation time ↑ Anti-cancer effect	[74]
Letrozole	Drug-related side effects	Poly (D, L-lactide) (PDLLA)	Emulsion-solvent evaporation	Study not done	Enhanced solubility	[75]
Curcumin	Poor solubility	PVP	Organic phase synthesis (Double- or single-phase process)	MDA-MB-231 MCF-7, L929 and MCF 10A	Anti-tumor and Inhibitory effect	[76]
Curcumin	Poor solubility	Chitosan	By modifying chitosan	MDA-MB-231	↑ Solubility ↑ Stability ↑ Cytotoxic effect	[77]
Curcumin	Low solubility Poor bioavailability Low stability Rapid degradation and metabolism	Alginate/chitosan	O/W emulsification and ionotropic gelation	MDA-MB-231 cells	Higher cytotoxicity Increased cellular uptake Enhanced stability study	[78]
Curcumin and Methotrexate	Low solubility Stability issues	PLGA	Double emulsion solvent evaporation technique	MDA-MB-231 and MCF-7 cell lines	↓ Proliferation of tumor, Synergistic effect and cytotoxic effect	[79]
Curcumin and Gemcitabine	Low solubility Adverse effects	PLGA	Double emulsion, a solvent evaporation technique	MDA-MB-231	Safer, higher and efficacious approach	[80]

**Table 6:** Various chemotherapeutic drugs and phytoconstituents and their combination polymeric nanoparticles for breast cancer treatment.

DRUGS	LACUNA ASSOCIATED	POLYMER USED	METHOD OF PREPARATION	IN VIVO/CELL LINE STUDY	OUTCOME	REFERENCES
Nimbolide	Poor solubility	PLGA	Nanoprecipitation	AsPC-1 and MCF-7 and MDA-MB-231	Enhanced cytotoxic effect ↑ Solubility	[81]
Disulfiram	Poor solubility	PLGA, PEG	Nanoprecipitation	MCF-7	Drug internalization into cell membrane Anticancer effect ↑	[82]
Suramin and Doxorubicin	Low solubility	Chitosan	Ionic gelation technique	TNBC model	Induction of angiogenesis	[83]
Nimesulide	Low solubility	Polymer-PEG-b-PCL	Emulsion-solvent evaporation, Nanoprecipitation	MCF-7	↑ Anticancer effects Pro-apoptotic effect	[84]
Piceatannol	Low bioavailability	Chitosan	Dropping method	MCF-7, A549, and HepG2	Mitochondrial dependent apoptosis	[85]
Artemisinin	Drug linked adverse effects	Chitosan	Precipitation method	4T1	Rapid drug release Enhanced therapeutic index	[86]
Rapamycin and Piperine	Low solubility Drug-related side effects	PLGA	Nanoprecipitation method	MDA-MB-231	Improved drug absorption Enhanced anticancer efficacy	[87]
Honokiol	Neurotoxic effects	PEG, PLGA	Nanoprecipitation method	EAC and MCF-7	Hindered proliferation of tumor Increased anticancer potential	[88]
Ormeloxifene	Systemic toxicity	PEGylated chitosan	Ionotropic gelation method	MCF-7 and MDA-MB-231	Increased cellular uptake, apoptosis, Stimulation of caspase-3, Destruction of mitochondria, Cytotoxicity in a dose-dependent way	[89]
Psoralen	Drug-related side effects Multi-drug resistance	PLGA	Emulsification evaporation-low temperature solidification	MCF-7, 7/ADR	Enhanced anti-cancer efficacy	[90]
Niclosamide	Poor water solubility	PLGA	Nanoprecipitation method	MCF-7, A549	Enhanced anti-cancer efficacy	[91]
Etoposide and Quercetin	Poor water solubility	PLGA	Single emulsification (o/w) solvent evaporation technique	MCF-7	Minimize the adverse effects, Sensitize the resistant cancerous cells Enhance the bioavailability	[92]
Evodiamine	Poor water solubility Drug-related side effects	PLGA	Single emulsion (o/w) solvent evaporation technique	MCF-7	Downregulate the β-actin expression and upregulate the cyclin B1 expression, Improved bioavailability	[93]
Sclareol	Poor aqueous solubility	PLGA	Nanoprecipitation method	MCF-7 and MDA-MB468	Enhanced stability Enhanced antitumor effect	[94]

**Table 7:** The various polymeric nanoparticles-based medicines approved by USFDA with their timeline of approval [98,99].

Brand Name	Company Name	Generic Name	Indication	Advantages	Approval Year
Oncaspar	Enzon Pharma	Aspargase	Acute lymphoblastic leukemia	Longer circulation time	1994
Copaxone	Teva	Glatimer acetate	Multiple sclerosis	Better clearance	1996
Neulasta	Amgen	Pegfilgrastim	Chemotherapy-induced neutropenia	Greater stability	2002
Pegasys	Genentech	Pegylated IFN alpha-2a	Hepatitis B, hepatitis C	Greater stability	2002
Somavert	Pfizer	Pegvisomant	Acromegaly	Greater stability	2003
Eligard	Tolmar	Leuprolide acetate	Prostate cancer	Longer circulation time and controlled drug release	2004
Renvela and Renagel	Genzyme	Sevelamer carbonate; and Sevelamer HCl	Chronic kidney disease	Longer circulation time	2007
Cimzia	UCB	Certolizumab pegol	Crohn’s disease, arthritis	Longer circulation and greater stability	2008
Plegridy	Biogen	Pegylated IFN beta-1a	Multiple sclerosis	Greater stability	2014
Adynovate	Shire	Antihemophilic factor	Hemophilia	Longer half-life and greater stability	2016
Rebinyn	Novo Nordisk	Coagulation factor IX	Hemophilia B	Longer circulation	2017
Mircera	Vifor	Methoxy polyethylene glycol-epoetin beta	Anemia associated with chronic kidney disease	Greater stability	2018
Zilretta	Flexion Therapeutics	Triamcinolone acetonide	Osteoarthritis knee pain	Extended drug release	2018

## Data Availability

This study did not report any data.
